# Pharmacological and optical activation of TrkB in Parvalbumin interneurons regulate intrinsic states to orchestrate cortical plasticity

**DOI:** 10.1038/s41380-021-01211-0

**Published:** 2021-07-28

**Authors:** Frederike Winkel, Maria Ryazantseva, Mathias B. Voigt, Giuliano Didio, Antonia Lilja, Maria Llach Pou, Anna Steinzeig, Juliana Harkki, Jonas Englund, Stanislav Khirug, Claudio Rivera, Satu Palva, Tomi Taira, Sari E. Lauri, Juzoh Umemori, Eero Castrén

**Affiliations:** 1 Neuroscience Center, HiLIFE, University of Helsinki, Helsinki, Finland; 2 Molecular and Integrative Biosciences Research Programme, University of Helsinki, Helsinki, Finland; 3 Faculty of Psychology and Neuroscience, Maastricht University, Maastricht, Netherlands; 4 Department of Veterinary Biosciences and Neuroscience Center, University of Helsinki, Helsinki, Finland

**Keywords:** Neuroscience, Molecular biology

## Abstract

Elevated states of brain plasticity typical for critical periods of early postnatal life can be reinstated in the adult brain through interventions, such as antidepressant treatment and environmental enrichment, and induced plasticity may be critical for the antidepressant action. Parvalbumin-positive (PV) interneurons regulate the closure of developmental critical periods and can alternate between high and low plasticity states in response to experience in adulthood. We now show that PV plasticity states and cortical networks are regulated through the activation of TrkB neurotrophin receptors. Visual cortical plasticity induced by fluoxetine, a widely prescribed selective serotonin reuptake inhibitor (SSRI) antidepressant, was lost in mice with reduced expression of TrkB in PV interneurons. Conversely, optogenetic gain-of-function studies revealed that activation of an optically activatable TrkB (optoTrkB) specifically in PV interneurons switches adult cortical networks into a state of elevated plasticity within minutes by decreasing the intrinsic excitability of PV interneurons, recapitulating the effects of fluoxetine. TrkB activation shifted cortical networks towards a low PV configuration, promoting oscillatory synchrony, increased excitatory-inhibitory balance, and ocular dominance plasticity. OptoTrkB activation promotes the phosphorylation of Kv3.1 channels and reduces the expression of Kv3.2 mRNA providing a mechanism for the lower excitability. In addition, decreased expression and puncta of Synaptotagmin2 (Syt2), a presynaptic marker of PV interneurons involved in Ca^2+^-dependent neurotransmitter release, suggests lower inputs onto pyramidal neurons suppressing feed-forward inhibition. Together, the results provide mechanistic insights into how TrkB activation in PV interneurons orchestrates the activity of cortical networks and mediating antidepressant responses in the adult brain.

## Introduction

Brain plasticity is a key process allowing learning throughout life and adjustment of maladapted networks underlying neuropsychiatric diseases. The brain is particularly plastic during critical periods of early postnatal life, followed by a transition to a state of more limited plasticity. However, recently it became apparent that the adult brain can still operate in different states of plasticity induced by interventions, such as antidepressant treatment and environmental enrichment [1–3]. Chronic treatment with the antidepressant fluoxetine, for example, induces juvenile-like plasticity (iPlasticity) in the adult brain, resulting in a shift of ocular dominance (OD) after monocular deprivation [1] and fear erasure when combined with extinction training [2].

Experiments using the paradigm of OD plasticity in the primary visual cortex suggested that the maturation of GABAergic parvalbumin-expressing (PV) interneurons is a key determinant of brain plasticity states [4]. Toward the end of critical periods, PV interneurons are preferentially encased by perineuronal nets (PNNs) [5], extracellular matrix components rich in chondroitin sulfate proteoglycans [6]. PNN removal through enzymatic digestion during adulthood reinstates iPlasticity and reduces intracortical inhibition [5, 7] and this effect is mediated by the activation of TrkB in PV interneurons [8]. Furthermore, PV expression itself is plastic and regulated by experience during adulthood [9–11], and external experiences can switch the configurations of PV interneurons between plastic/immature and consolidated states as defined by low and high PV expression, respectively [9].

Brain-derived neurotrophic factor (BDNF) and its receptor Neurotrophic receptor tyrosine kinase 2 (Ntrk2, TrkB) are critical regulators of neuronal plasticity [12]. Antidepressant drugs directly bind to TrkB and increase BDNF signaling, which is critical for the cellular and behavioral effects of antidepressants [2, 13]. Furthermore, PV interneuron maturation and the closure of critical periods are promoted by BDNF signaling [14]. However, TrkB receptors are expressed in almost all neurons and it is not clear which neurons and mechanisms mediate the effects of TrkB in antidepressant action and cortical maturation.

Here, we use heterozygous conditional TrkB knockout mice and targeted TrkB activation to show that activation of TrkB in cortical PV interneurons is critical for the regulation of cortical plasticity and antidepressant responses. Low expression of TrkB in PV interneurons prevents an OD shift and induction of LTP observed in wild-type mice after chronic fluoxetine treatment. Experiments with a photoactivatable TrkB (optoTrkB) that can be activated in a spatially and temporally controlled manner [15, 16] confirm the importance of TrkB activation in PV interneurons for OD plasticity. Our results demonstrate that TrkB activation in PV interneurons dynamically regulates the intrinsic properties of PV interneurons by decreasing their intrinsic excitability and switching the PV network into a plastic configuration, which orchestrates adult cortical plasticity states and mediates the effects of antidepressants on neuronal plasticity.

## Materials and methods

Details of Material and Methods are in Supplementary information.

### Mice

Heterozygous mice with TrkB knocked out conditionally in PV^+^ interneurons (hPV-TrkB CKO mice), and mice expressing cre-recombinase in PV interneurons (PV-cre) were used. For patch-clamp electrophysiology, hPV-TrkB CKO and control mice were crossed with a TdTomato reporter line [17]. All mice were kept under 12 h light/dark cycle with light on at 6 a.m. and ad libitum access to food and water. All animal procedures were done according to the guidelines of the National Institutes of Health Guide for the Care and Use of Laboratory Animals and were approved by the experimental Animal Ethical Committee of Southern Finland (#ESAVI/10300/04.10.07/2016; ESAVI/38503/2019/Osahanke1).

### Fluoxetine treatment

Fluoxetine (Bosche Scientific, NJ, USA) was administered via drinking water (0.08 mg/ml), corresponding to a dose of ~8–12 mg/kg/day, and kept in light-protected bottles. The drinking water of both the control and fluoxetine group contained 0.1 % saccharin, was changed twice a week and the consumption was monitored.

### Construction of optoTrkB

Photolyase homology region domain of optoTrkB [15] was optimized in codon usage for mice and connected with a flexible tag [18] to the C-terminus of a full-length mouse TrkB, as previously described [19]. For cre-dependent expression of optoTrkB, double-floxed inverted open-reading frame (DIO) structure [16] of optoTrkB (DIO-optoTrkB) (Supplementary Fig. 1a) as well as that expressing TdTomato [20] (DIO-optoTrkB-IRES-TdT) (Supplementary Fig. 1c) were constructed.

### Virus generation and injection

Lentivirus expressing DIO-optoTrkB/DIO-optoTrkB-IRES-TdT were produced as previously described [21]. For lentivirus infection, PV-cre mice (3–5 or 7–11 months of age) were anesthetized with isoflurane and DIO-optoTrkB was stereotaxically injected into the binocular area of the V1.

### Ocular dominance plasticity paradigm

**Fig. 1:**
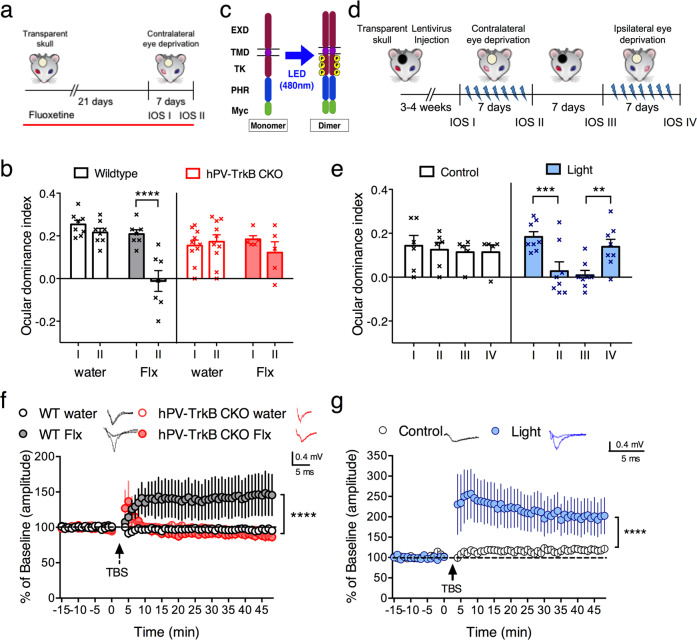
TrkB signaling in PV interneurons regulates visual cortex plasticity. **a** Experimental timeline of the shift in ocular dominance paradigm after fluoxetine (Flx) treatment. **b** ODI after chronic Flx treatment in WT and hPV-TrkB CKO mice. Flx permits MD to shift the ODI towards the non-deprived eye in V1 of WT mice, but fails to do so in hPV-TrkB CKO mice (IOS I vs II: WT water, *n* = 8, *p* = 0.6085; WT Flx, *n* = 7, *p* < 0.0001; CKO water, *n* = 10, *p* = 0.6326; CKO Flx, *n* = 5, *p* = 0.5727, two-way ANOVA with Holm-Sidak’s post-hoc test). **c** Structure of optoTrkB conjugated to a light reactive PHR domain, which promote dimerization optoTrkB upon blue light exposure. **d** Experimental timeline of the shift in ocular dominance paradigm with optoTrkB. OptoTrkB was stimulated with blue light twice daily (30 s) for 7 days during MD. **e** MD of the contralateral eye induces a shift in ocular dominance when combined with stimulation of optoTrkB (IOSI vs II: control, *n* = 6, *p* = 0.9950; light, *n* = 8, *p* = 0.0046). The shift is preserved if the eyes are not deprived (IOS II vs III: control, *p* = 0.9957; light, *p* = 0.6730, two-way ANOVA with Holm-Sidak’s post-hoc test). Stimulation of optoTrkB reverses shift in ocular dominance when combined with MD of the ipsilateral eye (IOS III vs IV: control, *n* = 5, *p* > 0.9999; light, *n* = 8, *p* = 0.0201, two-way ANOVA with Sidak’s post-hoc test). One mouse excluded due to corneal infection. **f** LTP recordings from layer II/III in V1 of WT and hPV-TrkB CKO mice after Flx treatment. TBS induces LTP only in WT mice treated with Flx (WT water (*n* = 6) vs. WT Flx (*n* = 10), *p* ≤ 0.0001; CKO water (*n* = 9) vs. CKO Flx (*n* = 10), *p* = 0.5916, Two-way ANOVA with Holm-Sidak’s post-hoc test). The VC in sections were randomly recorded from three animals in each group. **g** LTP recordings from layer II/III in V1. TBS results in LTP in slices where optoTrkB has been activated for 30 s 30 min prior to LTP induction (*n* = 6), but not in control slices kept in darkness (*n* = 7) (two-way ANOVA, light, *p* < 0.0001). The VC in sections were randomly recorded from six animals. Bars represent means ± SEM. *p < 0.05; **p < 0.01; ***p < 0.001; ****p < 0.0001.

For chronic imaging of intrinsic signals, the animals underwent transparent skull surgery and analysis was performed as previously described [22]. Fluoxetine was administered for 3 weeks in drinking water and the treatment was continued during monocular deprivation; controls received tap water (Fig. 1a). In the case of optoTrkB experiments, after the stereotaxic virus infection, the transparent skulls were immediately painted black and the paint was only transiently removed during the light stimulation and imaging (Fig. 1d). For monocular deprivation (MD), the animals were anesthetized with isoflurane, the eye lashes were trimmed and the eyelid margins were sutured shut. The eyes were checked daily until reopening, resutured if needed, and mice with signs of corneal injury were excluded from the experiments. For optical stimulation, black paint was removed and the visual cortex of optoTrkB-transfected animals was stimulated by blue light (470 nm) through the transparent skull [22] twice daily for 30 s during 7 days of MD; skulls were kept re-painted between stimulation sessions. OptoTrkB infected mice that had their transparent skull painted black all times and not stimulated by light served as controls. For optical imaging of intrinsic signals, we determined the strength of neuronal responses to stimulation of either eye in the binocular region of the V1 using imaging of intrinsic signals (IOS) [23] before (IOS I; IOS III) and after (IOS II; IOS IV) MD in anaesthetized mice. IOS responses were recorded from V1 [23] and modified for the measurement of OD plasticity [24]. The ocular dominance index [ODI: (C−I)/(C + I), where “C” and “I” refer to the response magnitude of the contralateral and the ipsilateral eye, respectively] was then calculated for every pixel within the binocularly responding region.

### In vivo electrophysiology

Local field potentials (LFP) from the binocular region of the optoTrkB transfected area of V1 of anesthetized mice were measured with a 16 channel optrode (A1x16-10 mm-100-177-OA16LP, NeuroNexus, Ann Arbor, MI) at a sampling rate of 20 kHz, using a Smartbox (NeuroNexus, Ann Arbor, MI). Neuronal oscillations between 4 and 112 Hz were extracted by Morlet-wavelet filtering after normalization and current source density analysis of the LFP data.

### Electrophysiology in acute slices

Field excitatory postsynaptic currents recording, and patch clamp recordings from pyramidal cells and TdT expressing PV interneurons were performed within layer II/III of the V1. Synaptic responses were evoked by electric stimulation at the border of the white matter (WM) and layer VI. All experiments using slices expressing optoTrkB were conducted in darkness. The brains were dissected, cut and stored as previously described [25]. WinLTP (www.winltp.com) was used for data acquisition and analysis.

### Statistical analysis

Normally distributed data were analyzed parametrically with unpaired *t* test (two-sided), two-way and one-way ANOVA followed by Holm-Sidak’s post hoc tests. Otherwise data were analyzed nonparametrically, such as Kruskal-Wallis followed by Dunn’s multiple comparison test. All statistics were performed using Graphpad Prism v.6.07. The significance level was set to 0.05 (*P* value).

## Results

### TrkB signaling in PV interneurons regulates visual cortex plasticity

Using the standard protocol for ocular dominance plasticity (OD), we confirmed that 7 days of monocular deprivation (MD) induced a shift of OD in the primary visual cortex (V1) of adult wild-type (WT) mice chronically treated with fluoxetine for three weeks but not in water-treated controls, as described previously [1, 22]. However, no OD shift was induced in mice heterozygous for PV-specific conditional TrkB knockout (hPV-TrkB CKO) (Fig. 1a, b). These data suggest that TrkB signaling specifically in PV interneurons is required for OD plasticity induced by chronic fluoxetine treatment.

TrkB is expressed in essentially all cortical neurons and BDNF can activate TrkB receptors in all of them. To specifically activate TrkB in PV interneurons, we infected the V1 of adult PV-cre mice with a lentivirus expressing DIO-optoTrkB (Fig. 1c) (Supplementary Fig. 1a, b). An acute 30 s light stimulation of optoTrkB in V1 resulted in increased phosphorylation of TrkB and CREB (Supplementary Fig. 2), indicating successful activation of optoTrkB and downstream signaling in vivo. We then stimulated the V1 of DIO-optoTrkB infected mice through a transparent skull [22] twice daily for 30 s by blue light during 7 days of MD (Fig. 1d, Supplementary Table 1) and observed a shift in ocular dominance index (ODI) toward the non-deprived ipsilateral eye (Fig. 1e). The transparent skulls were kept painted black all time after the infections, except during the light exposures. The shift persisted for a week in absence of visual deprivation and light stimulation (Fig. 1e). We then closed the ipsilateral eye that was previously left open, exposed V1 to light, and again observed a shift towards the non-deprived eye (Fig. 1e). In contrast, mice infected with DIO-optoTrkB but having their transparent skull painted black to prevent light exposure failed to show any OD plasticity (Fig. 1e). Light stimulation alone without optoTrkB infection during 7 days of MD induced no shift (Supplementary Fig. 3a). Together, these data indicate that TrkB activation in PV interneurons is necessary and sufficient for iPlasticity in the adult V1.

A shift in OD requires structural changes and takes several days to occur but we hypothesized that a change in plasticity state could be observed earlier. We, therefore, studied theta-burst stimulation (TBS)-induced long-term potentiation (LTP), which in acute V1 slices is normally restricted to critical periods [26]. As demonstrated before, LTP was successfully induced by TBS stimulation after chronic fluoxetine treatment [1] (Fig. 1f). In slices from hPV-TrkB CKO mice, however, TBS stimulation failed to induce LTP after fluoxetine treatment (Fig. 1f).

Strikingly, TBS stimulation induced a robust LTP in slices from mice infected with optoTrkB and acutely activated with a single 30 s light stimulation 30 min prior to TBS stimulation, indicating that TrkB activation in PV interneurons can rapidly alter the network state to permit LTP. No LTP was observed in optoTrkB infected slices kept in darkness (Fig. 1g).

### TrkB activation regulates PV-plasticity

**Fig. 2:**
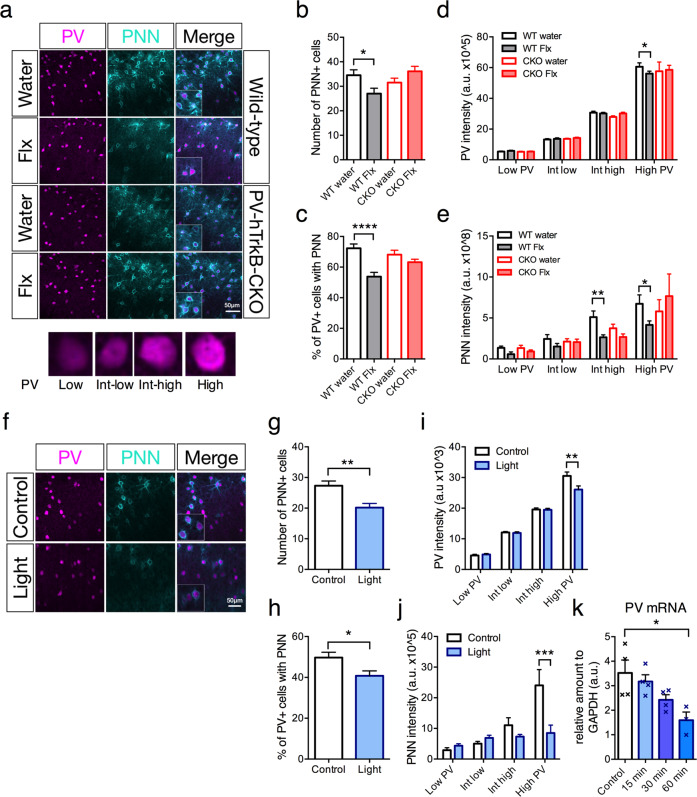
TrkB activation regulates PV-plasticity. **a** Representative immunohistochemical images of PV and PNN expression in layer II/III of Flx-treated WT and hPV-TrkB CKO mice. (lower panel) Representative images of low, intermediate-low, intermediate-high, and high PV expressing cells, where PV interneurons are categorized according to PV intensity. **b** Flx-treatment decreases numbers of PNN in WT but not hPV-TrkB CKO mice (Water vs flx: WT, *p* = 0.0302, CKO, *p* = 0.1537, one-way ANOVA with Holm-Sidak’s post-hoc test). **c** Flx-treated WT mice have significantly lower percentages of PV interneurons also expressing PNNs, but this effect is abolished in hPV-TrkB CKO mice (water vs Flx: WT, *p* < 0.0001; CKO, *p* = 0.2458, one-way ANOVA with Holm-Sidak’s post-hoc test). Number of images of visual cortex: Wt, Water, *n* = 19; Wt, flx, *n* = 12; CKO, water, *n* = 17; CKO, flx, *n* = 11, taken from 8, 7, 10, and 5 mice, respectively. **d** Flx treatment decreases PV intensities in high PV expressing cells in WT but this effect is not seen in hPV-TrkB CKO mice (water vs Flx: WT, p = 0.0420; CKO, *p* = 0.9181, two-way ANOVA with Holm-Sidak’s post-hoc test). **e** Flx treatment reduces PNN intensities in intermediate-high and high PV expressing cells only in WT mice but not in hPV-TrkB CKO mice (water vs Flx: WT, intermediate-high, *p* = 0.0008; high PV, *p* = 0.0173; CKO, intermediate-high, *p* = 0.3297; high PV, *p* = 0.5438, two-way ANOVA with Holm-Sidak’s post-hoc test). Number of cells: Wt, Water, *n* = 168; Wt, flx, *n* = 157; CKO, water, *n* = 178; CKO, flx, *n* = 162, were randomly selected from 8, 7, 10, and 5 mice, respectively. **f** Representative immunohistochemical images of PV and PNN expression in layer II/III of V1. Extended light stimulation of optoTrkB significantly reduced numbers of PNN positive cells (*p* = 0.0012, Unpaired *t* test) **g** and percentages of PV interneurons also expressing PNNs (*p* = 0.0156, Unpaired *t* test). **h** Number of images: control, *n* = 23; light, *n* = 23, were taken from 6 and 8 mice, respectively. **i** Stimulation of optoTrkB significantly reduces the PV staining intensities of high PV expressing cells (*p* = 0.0003, Two-way ANOVA with Holm-Sidak’s post-hoc test). Number of cells: control, *n* = 98; light, *n* = 101, were randomly selected from 6 and 8 mice, respectively. **j** Activation of optoTrkB decreases PNN intensities in high PV expressing cells (*p* = 0.0003, Two-way ANOVA with Holm-Sidak’s post-hoc test comparing light-stimulated vs. control samples). **k** qPCR measurements of PV mRNA with control (non-stimulated, *n* = 4), 15 min (*n* = 4), 30 min (*n* = 4) and 60 min after light stimulation with V1 tissue. OptoTrkB reduces the expression of PV mRNA 60 min after light stimulation (One-way ANOVA, *p* = 0.0186; Holm-Sidak’s post-hoc test, control vs. 60 min, *p* = 0.0124). Bars represent means ± SEM. *p < 0.05; **p < 0.01; ***p < 0.001; ****p < 0.0001.

We hypothesized that elevated V1 plasticity could also be accompanied by PV and PNN plasticity, and conducted immunohistochemistry with PV antibody and ﻿Wisteria floribunda agglutinin (WFA) staining for PNNs [27] (Fig. 2a). Chronic fluoxetine treatment reduced the numbers of PNN-positive (PNN^+^) cells (Fig. 2b) and PV interneurons encased by PNN^+^ (Fig. 2c) as previously reported in amygdala [2]. We then measured the expression intensities of PV as described previously [9] but included the relation to PNN intensities. We observed a reduction in PV and PNN intensities specifically in high PV, and high and intermediate-high PV expressing interneurons, respectively (Fig. 2d, e), indicating the regression of PNNs particularly in the PV cell population expressing higher amounts of PV. These effects, however, were not detected in hPV-TrkB CKO mice (Fig. 2a-e).

We then verified whether optoTrkB stimulation exerts similar effects (Fig. 2f). Consistently, optoTrkB stimulation of visual cortical PV interneurons (30 s stimulation once daily for 7 days) reduced the numbers of PNN^+^ (Fig. 2g) and PNN^+^ within the PV population (Fig. 2h). In addition, optoTrkB activation decreased PV and PNN intensities in high PV expressing cells (Fig. 2i, j). Interestingly, we also found a decrease in PV mRNA expression 60 min after optoTrkB stimulation, strongly supporting that optoTrkB dynamically regulates PV expression. These results indicate that pharmacological and optogenetic activation of TrkB shifts PV and PNNs to plastic configurations, especially in PV interneurons with consolidated states characterized by higher PV expression.

### TrkB activation in PV interneurons reduces cell excitability and EPSC frequencies

**Fig. 3:**
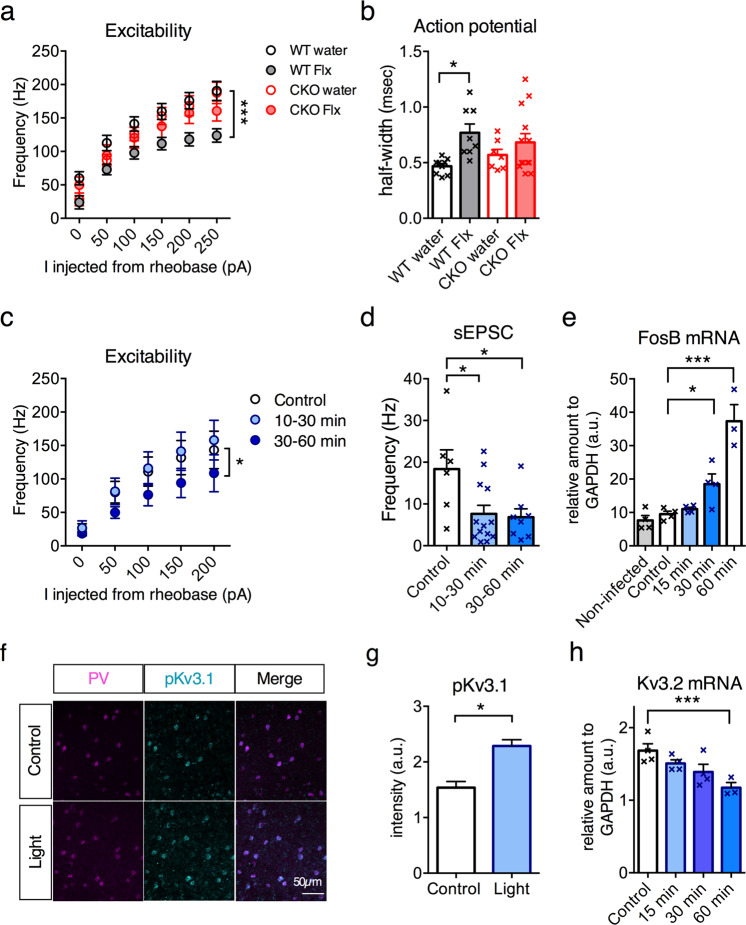
TrkB activation in PV interneurons reduces cell excitability. **a** Whole-cell patch-clamp analysis of PV interneurons in WT and hPV-TrkB CKO mice after chronic Flx treatment. The intrinsic excitability is reduced after Flx treatment in WT mice but not in hPV-TrkB CKO mice (WT water vs. WT Flx, *p* < 0.0001; CKO water vs. CKO Flx, *p* = 0.2851, Two-way ANOVA with Holm-Sidak’s post-hoc test). Number of cells: Wt, Water, *n* = 9; Wt, flx, *n* = 8; CKO, water, *n* = 7; CKO, flx, *n* = 13, were randomly recorded from three animals in each group. Outliers were excluded after ROUT analysis (*Q* = 1%) **b** Flx treatment increases AP half-width in WT mice but not in hPV-TrkB CKO mice (WT water vs. WT Flx, *p* = 0.0102; CKO water vs. CKO Flx, *p* > 0.9999, Kruskal–Wallis test with Dunn’s post-hoc test). Number of cells: Wt, Water, *n* = 9; Wt, flx, *n* = 8; CKO, water, *n* = 7; CKO, flx, *n* = 13, randomly selected from three animals in each group. **c** Whole-cell patch-clamp recordings of intrinsic excitability of optoTrkB-infected PV interneurons. Activation of optoTrkB decreases intrinsic excitability of PV interneurons at 30–60 min after light stimulation (Two-way ANOVA, activation, *p* = 0.0385). Number of cells: control, *n* = 6; 10–30 min after light exposure, *n* = 8; 30–60 min after light exposure, *n* = 6; slices were randomly stimulated/non-stimulated and recorded from 10 mice. Outliers were excluded after ROUT analysis (*Q* = 1%). **d** sEPSC frequency in PV interneurons is decreased at 10–30 min after light stimulation as compared to controls. (10–30 min, *p* = 0.0232; 30–60 min, *p* = 0.0232, one-way ANOVA with Holm-Sidak’s post-hoc test). Number of cells: control, *n* = 6; 10- 30 min after light exposure, *n* = 13; 30–60 min after light exposure, *n* = 8; slices were randomly stimulated/non-stimulated and recorded from ten mice. **e** qPCR quantification of FosB mRNA of non-infected (*n* = 4), control (non-stimulated, *n* = 4), 15 min (*n* = 4), 30 min (*n* = 4) and 60 min (*n* = 3) after light stimulation. The mRNA levels are increased 60 min after light stimulation (one-way ANOVA, *p* < 0.0001; Holm-Sidak’s post hoc, *p* < 0.0001). **f** Representative images of PV, phospho-Kv3.1 and merged immunohistochemistry staining. **g** Light stimulation of optoTrkB results in increased intensity of phospho-Kv3.1 staining in PV interneurons in layer II/III of V1 (Unpaired *t* test. Number of cells, *n* = 82 from six animals in each group. 7 days twice daily light stimulation). **h** qPCR quantification of Kv3.2 mRNA after light stimulation. mRNA levels are decreased 60 min after light stimulation in Kv3.2 (one-way ANOVA, *p* = 0,0128, Holm-Sidak’s post hoc, control vs 60 min, *p* = 0.0059). Control (non-stimulated, *n* = 4); 15 min (*n* = 4); 30 min (*n* = 4) and 60 min (*n* = 3) after light stimulation. Bars represent means ± SEM. *p < 0.05; **p < 0.01; ***p < 0.001; ****p < 0.0001.

To investigate intrinsic changes, we obtained patch-clamp recordings from PV interneurons expressing TdTomato as a reporter in WT and hPV-TrkB CKO mice. Chronic fluoxetine treatment in WT mice reduced the intrinsic excitability of PV interneurons (Fig. 3a) and increased action potential (AP) half-width (Fig. 3b) without any changes in sEPSC and sIPSC frequencies or amplitudes (Supplementary Fig. 4a–e). In hPV-TrkB CKO mice, however, fluoxetine failed to induce any alterations in intrinsic properties of PV interneurons (Fig. 3a, b, Supplementary Fig. 4a-e). The AP width of CKO mice showed a relatively high variation, which may reflect variation in the TrkB expression levels in PV interneurons of some heterozygous mice. These data suggest that the reduced excitability by fluoxetine treatment depends on expression of TrkB in PV interneurons (Supplementary Table 2).

We then tested if an acute activation of TrkB signaling in PV interneurons was sufficient to replicate the effects of chronic fluoxetine treatment by recording from optoTrkB-positive PV interneurons co-expressing TdTomato in V1 (Supplementary Fig. 1c, d). Strikingly, the intrinsic excitability of PV cells was lower 30–60 min after a 30 s light stimulation compared to non-stimulated controls (Fig. 3c). In addition, we observed a rapid decrease in sEPSC frequencies (Fig. 3d), however, no changes in sIPSC frequencies (Supplementary Fig. 4f–j, Supplementary Table 2). In fact, expression of FosB mRNA was increased in V1 tissue after optoTrkB activation in PV interneurons (Fig. 3e), indicating transcriptional response. Importantly, there was no difference in FosB expression in visual cortical samples between non-infected mice and mice infected but not exposed to light stimulation. Together with the comparable SOD between non-infected control (Fig. 1b) and optoTrkB-infected control mice (Fig. 1e), this suggests that viral expression of optoTrkB has no detectable baseline effects in the visual cortex. These results indicate that TrkB activation by chronic fluoxetine treatment and acute optogenetic stimulation similarly and dynamically down-regulate the intrinsic excitability of PV interneurons.

We next took advantage of the high temporal and spatial precision of optoTrkB to investigate the underlying mechanisms. Potassium currents are known to regulate intrinsic excitability of neurons and Kv3 channels are highly expressed in cortical PV interneurons, regulating the fast-spiking properties [28, 29]. Kv3.1 channels are directly inhibited by phosphorylation through PKC [30], a downstream target of TrkB signaling. We therefore immunohistochemically examined the expression intensities of phospho-Kv3.1 within PV interneurons, and found that optoTrkB activation increased phospho-Kv3.1 expression in PV interneurons in visual cortical slices (Fig. 3f, g). In addition, qPCR with V1 tissue samples of optoTrkB-infected mice showed a reduction in the expression of Kv3.2 mRNA (Fig. 3h) and a progressive trend toward reduction of Kv3.1 mRNA 60 min after light stimulation (Supplementary Fig. 3b). Enhanced phosphorylation and reduced expression of Kv3 channels might largely drive the decreased excitability of PV interneurons.

### Disinhibition of pyramidal neurons accompanied with reduced Syt2 expression via TrkB activation in PV interneurons

**Fig. 4:**
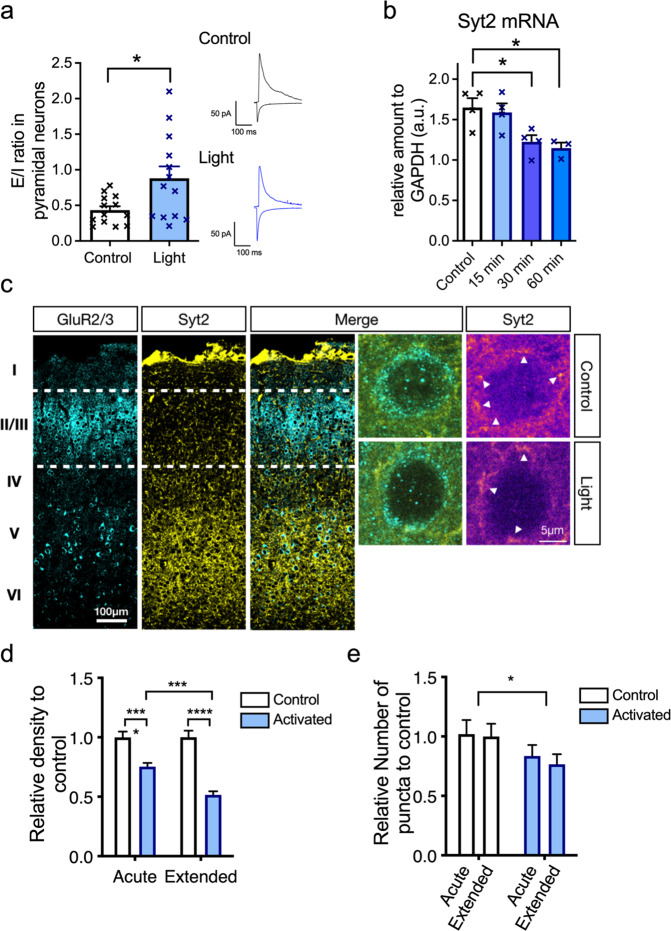
TrkB activation in PV interneurons results in disinhibition of pyramidal neurons. **a** Patch-clamp recordings from layer II/III pyramidal cells in V1 reveal an increase in excitation/inhibition ratio (E/I ratio; EPSC/disynaptic IPSC) after light activation of optoTrkB in PV interneurons (*p* = 0.0232, Unpaired *t* test with Welch’s correction).13 cells/13 slices (one cell per slice), 5 animals per group (light/control). Right panel shows representative traces of controls and optoTrkB-stimulated samples. **b** qPCR quantification of Syt2 mRNA. Levels of Syt2 mRNA are decreased 30 and 60 min after light stimulation (one-way ANOVA, *p* = 0.0105; Holm-Sidak’s post hoc analysis, *p* = 0.0208, and *p* = 0.0186, respectively). Control (non-stimulated, *n* = 4), 15 min (*n* = 4), 30 min (*n* = 4), and 60 min (*n* = 3) after light stimulation. **c** Immunohistochemistry with GluR2/3 and Syt2 in slices of V1 to analyze Syt2 density and puncta in GluR2/3 positive cells in layer II/III. **d** Relative expression of Syt2 was significantly decreased after acute and extended activation of optoTrkB compared to non-activated control (Two-way ANOVA, activation, *P* < 0.0001; Holm-Sidak’s post hoc, control vs activation, acute, *p* < 0.0001; extended, *p* < 0.0001). The expression was further reduced after extended activation compared to acute (Two-way ANOVA, time, *p* = 0.0109; Holm-Sidak’s post hoc, activated, acute vs extended, *p* = 0.0009). Number of cells: acute, control, *n* = 108; acute, activated, *n* = 84; extended, control, *n* = 59; extended, activated, *n* = 58, were randomly analyzed from 3, 4, 4, and 4 mice, respectively. **e** Number of puncta after optoTrkB activation was significantly reduced (Two-way ANOVA, activation, *p* = 0.0412). Number of cells: acute, control, *n* = 46; acute, activated, *n* = 58; extended, control, *n* = 46; extended, activated, *n* = 48, were randomly analyzed from 3, 4, 4, and 4 mice, respectively. Bars represent means ± SEM. *p < 0.05; **p < 0.01; ***p < 0.001; ****p < 0.0001.

To study whether TrkB-mediated changes in PV interneuron excitability regulates excitation/inhibition balance in the cortical microcircuitry, we investigated the strength of feed-forward inhibition in layer II/III pyramidal cells in visual cortical slices following light-activation of optoTrkB in PV interneurons. The ratio of monosynaptic excitatory to disynaptic inhibitory responses in pyramidal neurons was increased after stimulation of optoTrkB in PV interneurons (Fig. 4a), demonstrating reduced feed-forward inhibition onto pyramidal cells. To further investigate the mechanism underlying disinhibition of pyramidal neurons, we checked expression and number of puncta of Synaptotagamin2 (Syt2), a Ca^2+^ sensor that is involved in synaptic vesicle release in presynaptic inhibitory terminals of PV^+^ interneurons [31, 32]. The mRNA expression of Syt2 was reduced 30 min after optoTrkB activation in PV^+^ interneurons (Fig. 4b). Remarkably, immunohistochemical analysis showed that the intensity of Syt2 was significantly reduced after acute activation of optoTrkB and further after extended one-week daily activation (Fig. 4c, d). The total number of puncta was also decreased (Fig. 4e). These results suggest that optoTrkB activation downregulated PV interneuron excitability and reduced inhibitory PV interneuron inputs onto pyramidal cells, which together contribute to network disinhibition.

### Network changes induced by TrkB activation in PV interneurons

**Fig. 5:**
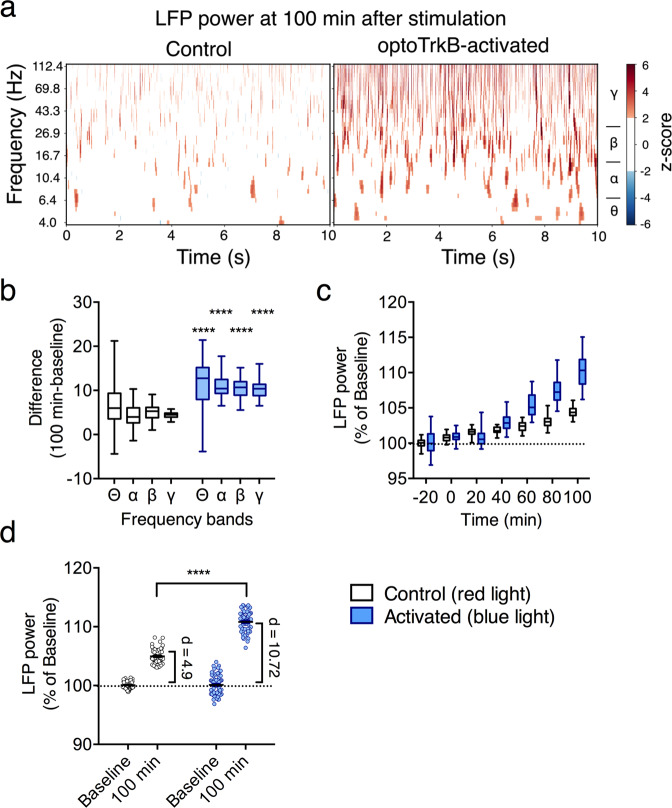
Network changes induced by optoTrkB activation in PV interneurons. **a** Wavelet spectra of red light-exposed (780 nm, control) and blue light-exposed (470 nm, optoTrkB-activated) mice expressing optoTrkB in PV interneurons 100 min after light stimulation. Color scale = Power (*z*-score). **b** Difference of LFP power between 100 min after light stimulation and baseline (black: control wavelength at 780 nm, blue: optoTrkB activated at 470 nm) in frequency bands (*θ*, *α*, *β*, and *γ*). The differences of LFP power in all bands are higher in blue light stimulation compared to red light stimulation (Two-way ANOVA, activation, *P* < 0.0001; Holm-Sidak’s post-hoc, control vs activated in *θ*, *α*, *β*, and *γ*: *p* < 0.0001. **c** Broadband LFP power averaged over animals for each 20 min recording session as a function of time after light stimulation. LFP power shows a significant increase over time (Regression analysis; Control: *y* = 0.04 [%/min]* x [min] + 100.25, *R*^2^ = 0.7573, *p* < 0.0001; Light: *y* = 0.09 [%/min]* x [min] + 101.01, *R*^2^ = 0.8240, *p* < 0.0001). **d** Broadband LFP power from baseline period (left plot of each half) and 100-min after blue light (470 nm) stimulation (right plot of each half). The LFP power was approximately twice as strong after optoTrkB activation (blue, difference in medians: *d* = 10.72) than after stimulation with control wavelength (780 nm) (black, difference in medians: *d* = 4.90). A Welch’s *t* test confirmed that the LFP power in the 100-min condition bin after optoTrkB activation is higher than after control stimulation (unpaired *t* test with Welch’s correction, *t* = 21.60, *p* < 0.0001). Control, *n* = 4; Activated, *n* = 5. Bars represent means ± SEM. ****p < 0.0001.

Plasticity in the visual cortex is controlled locally by synchronized neuronal oscillations, which are regulated by the activity of PV interneurons [33, 34]. In vivo local field potential (LFP) recordings of neuronal activity from V1 showed an increase in the magnitude of gamma (γ) range bands at 100 min after optoTrkB activation compared to baseline (Fig. 5a, b). Interestingly, the magnitudes of theta (θ), alpha (α), and beta (β) bands were also increased after the activation (Fig. 5b, Supplementary Fig. 5), leading to a general increase in broadband (4−112 Hz) LFP power in response to optoTrkB activation (Fig. 5c, d) when compared to the pre-stimulation baseline. Control stimulation with infrared light (780 nm) in optoTrkB infected animals did not influence oscillations (Fig. 5a–d). These results indicate that TrkB activation in PV interneurons promotes oscillatory synchrony to drive a state of elevated plasticity.

## Discussion

Previous studies show that chronic fluoxetine treatment reactivates OD plasticity in the adult visual cortex, which is driven by decreased intracortical GABAergic transmission and increased BDNF protein levels, suggesting the involvement of the interneuron network and TrkB signaling as the underlying mechanisms [1, 22]. Here, we show that plasticity induced by fluoxetine treatment in the visual cortex is dependent on TrkB signaling specifically in PV interneurons. Using optoTrkB that allows spatial and temporal specificity, we show that activation of TrkB in PV interneurons promotes plasticity and regulates PV and PNN configurations. TrkB activation-induced intrinsic changes in PV interneurons leading to network-level responses, such as increased excitation-inhibition balance, synchronized oscillations and visual cortex plasticity. Although PV interneurons cover only a small percentage of the total neuronal population, their extensive axonal arborization enable strong inhibitory control over pyramidal cells [35]. Strikingly, while BDNF-induced TrkB activation during critical periods promotes the maturation of PV interneurons [14], TrkB activation in PV interneurons during adulthood can reinstate a critical period-like plasticity by rejuvenating the interneurons.

### Differential effects of TrkB activation in pyramidal neurons and interneurons

iPlasticity is associated with reduced cortical inhibition and subsequent disinhibition of cortical networks [36]. BDNF has been shown to strongly promote neuronal activity, excitability [37–39], and LTP [12, 14] in hippocampal and cortical excitatory neurons. In contrast, we found that activation of TrkB in PV interneurons decreases excitability of PV cells, promoting LTP in V1. This suggests that activation of TrkB produces differential effects on excitability and electrophysiological properties depending on neuronal cell types. Importantly, however, from the point of view of cortical networks, these differential effects of BDNF/TrkB signaling on excitability cooperatively drive increased network activity.

### TrkB regulates PV plasticity

PV interneurons retain intrinsic plasticity and can alternate between high and low plasticity states when exposed to different external stimuli [9]. Environmental enrichment that is known to promote neuronal plasticity, induces a low PV expressing network state, whereas contextual fear conditioning associated with network consolidation shifts the PV network into a high PV expressing state [9]. PV expression progressively increases throughout development and could therefore also account for the reduction in brain plasticity during adulthood [9, 40]. Interestingly, pharmacological or optical TrkB activation in PV interneurons can regulate PV plasticity states, and this effect may be mediated through direct regulation of PV mRNA expression as confirmed by qPCR. As PV is a Ca^2+^ buffer, a low PV expressing state could also directly regulate PV interneuron firing [35, 41].

The maturation of the PV network is associated with the formation of PNNs [5]. In the hippocampus, PV intensity is generally higher in PV cells enwrapped by PNNs and PV cells with weak PV intensity often lack PNNs [42]. Enzymatic digestion of PNNs restores plasticity in adulthood [5, 7] and results in a decrease in PV intensity and mRNA levels, suggesting a correlation between PNN expression and PV configuration states [42]. A recent study demonstrates that transient electrical silencing of visual cortical PV interneurons induces a regression of PNNs [43], indicating that the activity state of PV interneurons affects PNN assembly and turnover. Our results suggest that TrkB activity within PV interneurons directly regulates PV expression and PNN levels, thereby contributing to the plasticity state of PV interneurons. However, whether the regression of PNNs is a cause or a consequence of reduced PV expression remains a subject for future studies.

### Activation of TrkB mediates intrinsic changes in PV interneurons

The hallmark of PV interneurons is their high-frequency firing, which is enabled by the high expression of voltage-gated Kv3 channels [29, 35]. Kv3 channels are characterized by their fast deactivation during membrane repolarization, enabling sustained high-frequency firing. The maturation of PV cells throughout critical period coincides with an increase in the fast-spiking firing frequency and an increase in Kv3.1 expression [28, 44]. A decrease in Kv3 channel expression, as well as their inhibition through PKC mediated phosphorylation, are known to reduce cell excitability [30, 45]. PKC is a downstream target of TrkB signaling [12] and TrkB activation in PV interneurons promotes enhanced phosphorylation of Kv3.1 and decreased expression of Kv3.1 and Kv3.2 channels, suggesting control of TrkB signaling over PV intrinsic excitability. Kv3 channels are specifically expressed in fast-spiking neurons, which could account for the differential effects of TrkB activation on excitability of pyramidal cells and PV interneurons.

Syt2 expression was recently shown to be positively correlated with the intensity of PNN and to cooperatively regulate the maturation state of inhibitory networks [46]. Our results imply that TrkB activation in PV interneurons plays a pivotal role in the regulation of genes involved in inhibitory network maintenance and connectivity. To capture the whole width of genes involved, we are currently analyzing single nuclei transcriptome after optoTrkB activation in PV interneurons.

In conclusion, we provide evidence that the plasticity effects of antidepressant treatment depend on TrkB signaling in PV interneurons. We further developed a tool to spatially and temporally control TrkB signaling in PV interneurons, which revealed that TrkB-mediated plasticity in PV interneurons is faster than previously anticipated. OptoTrkB also allowed us to investigate the mechanisms underlying the switch in plasticity configuration of PV cells, which dynamically orchestrates cortical networks. Although we used the visual cortex as a model network, these mechanisms might be extrapolated to other brain areas. Together with the recently discovered direct binding site for antidepressants in the TrkB dimer [13], these findings may enable a rational design of more specific and efficient clinical interventions for the treatment of mood disorders and other conditions that benefit from enhanced plasticity.

## Supplementary information

Supplemental information

Supplemental Figure 1

Supplemental Figure 2

Supplemental Figure 3

Supplemental Figure 4

Supplemental Figure 5

Supplemental Table 1

Supplemental Table 2
