# Supplementary material for: Pharmacological and optical activation of TrkB in Parvalbumin interneurons regulate intrinsic states to orchestrate cortical plasticity
Source: Mol Psychiatry. 2021 Jul 28;26(12):7247–56. doi: 10.1038/s41380-021-01211-0 (PMC8872988; doi:10.1038/s41380-021-01211-0)
Supplement: Supplementary file 1 — Supplemental information [file 41380_2021_1211_MOESM1_ESM.docx]

# **Pharmacological and optical activation of TrkB in Parvalbumin interneurons regulates intrinsic states to orchestrate cortical plasticity**

Frederike Winkel^1^, Maria Ryazantseva^1,2^, Mathias B. Voigt^1^, Giuliano Didio^1^, Antonia Lilja^3^, Maria Llach Pou^1^, Anna Steinzeig^1^, Juliana Harkki^1^, Jonas Englund^1,2^, Stanislav Khirug^1^, Claudio Rivera^1^, Satu Palva^1^, Tomi Taira^1,4^, Sari E. Lauri^1,2^, Juzoh Umemori^1§^, Eero Castrén^1^

1 Neuroscience Center, HiLIFE, University of Helsinki, Finland.

2 Molecular and Integrative Biosciences Research Programme, University of Helsinki, Finland

3 Faculty of Psychology and Neuroscience, Maastricht University, Netherland

4 Department of Veterinary Biosciences and Neuroscience Center, University of Helsinki, Finland

§ Corresponding author

Juzoh Umemori,

Neuroscience center, HiLife, University of Helsinki, 00790 Helsinki, Finland

Phone +358-45-1578-930

[juzoh.umemori@helsinki.fi](mailto:juzoh.umemori@helsinki.fi)

**Material and Methods**

## **EXPERIMENTAL MODEL AND SUBJECT DETAILS**

All animal procedures were done according to the guidelines of the National Institutes of Health Guide for the Care and Use of Laboratory Animals and were approved by the experimental Animal Ethical Committee of Southern Finland (#ESAVI/10300/04.10.07/2016; ESAVI/38503/2019/Osahanke1). Both male and female mice were used and kept under 12h light/dark cycle with light on at 6 am and *ad libitum* access to food and water. For the shift in ocular dominance paradigm, the mice underwent transparent skull surgery and were monocularly deprived for 7 days.

## **Mice**

Heterozygous mice with TrkB deletion specifically in PV^+^ interneurons (PV-hTrkB mice; PV^pvr/wt^, TrkB^flx/wt^) were produced by mating females of heterozygous PV specific Cre line (PV^pvr/wt^) (Pvalb-IRES-Cre, JAX: 008069, Jackson laboratory) (1) and male of homozygous floxed TrkB mice (TrkB^flx/flx^) (2). Single floxed mice (TrkB^flx/wt^) served as control. For patch-clamp electrophysiology, females of the homozygous PV Cre line (PV^pvr/pvr^) were crossed with males harboring a homozygous TdTomato indicator allele (Rosa26^TdT/TdT^ ) (Ai14, JAX: 007914, Jackson laboratory, Bar Harbor, ME, USA)(3) and heterozygous floxed TrkB allele (TrkB^flx/wt^)(2) to reproduce compound transgenic mice with TrkB deletion and TdT expression specifically in PV interneurons (PV^pvr/wt^, TrkB^flx/wt^, Rosa26^TdT/wt^) (hPV-TrkB CKO) and wild-type allele in TrkB (PV^pvr/wt^, TrkB^wt/wt^, Rosa26^TdT/wt^) (WT). All of the parental strains were back-crossed with C57BL/6J for more than six generations. To assure the complete closure of the critical periods (4), the mice were 8-12 months old at the start of the experiments, except for patch clamp electrophysiology, where the mice ranged between 4-6 months of age. The ages among groups in the same experiments were equal. For shift of ocular dominance paradigm, only female mice (PV-cre, WT or hPV-TrkB CKO) were used, since male mice tend to start fighting after surgeries and need to be single housed, which might affect the outcome of experiments.

## **Construction of optoTrkB**

Photolyase homology region (PHR) domain of optoTrkB (5) was optimized in codon usage for mice and connected with a flexible tag (6) to the C-terminus of a full-length mouse TRKB, as previously described (Umemori et al, BioRxiv, 2021). For cre-dependent expression of optoTrkB, a double floxed inverted open-reading frame (DIO) structure (7) of optoTrkB (DIO-optoTrkB) was constructed (Figure S1A). We artificially synthesized DNA including human Synapsin promoter (8), lox2272 (Lee & Saito, 1998), loxP, inverted optoTrkB sequence [PHR, flexible tag (9), mouse full-length TrkB (NM_001025074)], lox2272, loxP, and cloned into pFCK(0.4)GW (a gift from Pavel Osten) (Addgene plasmid # 27229 ; <http://n>2t.net/addgene:27229; RRID:Addgene_27229) (10) using PacI and EcoRI cloning sites (Figure S1a). Furthermore, for electrophysiological studies, we constructed DIO-optoTrkB expressing TdTomato (11) (DIO-optoTrkB-IRES-TdT) (Figure S1C). A fragment of Internal ribosome entry site (IRES) with TdTomato was amplified by PCR with primers (5’- ggcgcgCCCCCCTCTCCCTCCCCCCC -3’ and 5’- ggcgcgccTTACTTGTACAGCTCGTCCATGCCGTACAG -3’) using Hot start Q5® polymerase (NEB, Frankfurt, Germany) from LeGO-iT (a gift from Boris Fehse) (Addgene plasmid # 27361 ; <http://n>2t.net/addgene:27361 ; RRID:Addgene_27361) (12), and cloned into pCR™Blunt II-TOPO® vector (ThermoFisher Scientific, Hvidovre, Denmark). Then the sequence was confirmed by sequencing and sub-cloning into AscI cloing sites of DIO-optoTrkB (Figure S1A) to construct DIO-optoTrkB-IRES-TdT. Cre-dependent Inversions of DIO-optoTrkB and DIO-optoTrkB-IRES-TdT were confirmed in HEK293 cells co-transfected with the DIO-vectors and plasmids expressing Cre recombinase followed by PCR (Set1 (Figure S1B), and immunocytology (Figure S1D). The following primers were used to confirm the inversion of DIO-optoTrkB:

Set1 (5’- AGTCGTGTCGTGCCTGAGA -3’ and 5’- GAAATTTATGTGCCGCAGGT -3’); Set2 (5’- CTGCTGGCAAAGGCTATTTC -3’ and 5’- GGGCCACAACTCCTCATAAA -3’).

## **Virus generation**

DIO-optoTrkB/DIO-optoTrkB-IRES-TdT, pLP1, pLP2, and pVSVG were co-transfected into HEL293FT cells by jetPEI® (Polyplus Transfection, Illkirch, France) according to the manufacturer’s instruction, for producing lentivirus through a previously reported method (13). Briefly, 3µg of the plasmids was transfected into HEL293FT cells and cultured on a poly-D-Lysine coated dish containing pre-warmed Opti-MEM® medium (ThermoFisher Scientific, Hvidovre, Denmark) with 2% FBS for 72 hrs. Then the culture supernatant was collected and exchanged with a new Opti-MEM® medium with 2% FBS followed by incubation for 72 hrs. The culture supernatant was collected again and centrifuged at 4 °C at 2000 g for 10 min. The supernatant was then concentrated with Amicon® Centrifugal Filter Units Ultra -15 (Merck Millipore, Darmstadt, Germany) into less than 10ml. The concentrated solution was purified using the sucrose gradient method (14) and aliquots were stored at -80 °C until use. From one aliquot the p24 capsid protein concentration was measured to estimate the infection unit (IU).

## **Surgery**

For chronic imaging of intrinsic signals and light exposure onto the VC, the animals underwent transparent skull surgery as described previously (15).

## **Lentiviral infection and optic stimulation of optoTrkB**

PV-cre mice were anaesthetized with 2.5% isoflurane and DIO-optoTrkB (p24 concentration/titer 4,04 x 10^7) was stereotaxically injected into the binocular area of the V1, previously identified through optical imaging (see below) and using blood vessels as landmarks. DIO-optoTrkB was stimulated with blue LED light (470nm) through the transparent skull during monocular deprivation twice a day for 30 seconds at 8-10 am and 4-6 pm. Blue LED light was provided by a BLS Super High Power Fiber-Coupled LED Light Sources (BLS-FCS-0470-100, Mightex, Pleasanton, CA) connected to a BioLED Light Source Control Modules (BLS-3000-2, Mightex, Pleasanton, CA). To avoid light exposure through the skull, the transparent skull was covered by black nail polish at all times after the infections. Black nail polish was removed only when the light was exposed onto transparent skull, and immediately covered by black nail polish after the exposure.

The intensity of blue light passing through an unpainted transparent skull was 44.4 mW/cmˆ2, which is clearly larger than 1.27mW cmˆ2 that has been shown to activate optoTrkB (5). In contrast, neither ambient light nor blue LED light reached the brain through a black-painted transparent skull (Supplementary Table 1), indicating no activation of optoTrkB when the transparent skulls were painted with black.

## **Fluoxetine treatment**

Fluoxetine (Bosche Scientific, New Brunswick, NJ) was administered via drinking water (0.08 mg/ml), corresponding to a dose of approximately 8-12 mg/kg/day, and kept in light-protected bottles. The drinking water of both the control and fluoxetine group contained 0.1 % saccharin, was changed twice a week and the consumption was monitored. Drug treatment started after transparent skull surgery and continued throughout the whole experiment.

## **Monocular deprivation and virus stimulation**

The animals were anaesthetized with intraperitoneal injection as described above. The eye lashes were trimmed and the eye lid margins were sutured shut with 3 mattress sutures. To prevent postoperative infections, an eye ointment containing dexamethasone was applied. The eyes were checked daily until reopening, re-sutured if needed, and mice with signs of corneal injury were excluded from the experiments.

## **Optical imaging of intrinsic signals**

We determined the strength of neuronal responses to stimulation of either eye in the binocular region of the V1 using imaging of intrinsic signals (IOS) (16) before (IOS I; IOS III) and after (IOS II; IOS IV) monocular deprivation. The animals were chamber anaesthetized with 1.8% isoflurane in a 1:2 mixture of O_2_/air and then intubated and ventilated with 1.2% isoflurane in the same mixture.

Intrinsic optical signal responses were recorded from the V1 of the right hemisphere according to a previously described protocol (16), which was modified for the measurement of OD plasticity (17).

## **Data analysis of optical imaging**

Cortical maps were computed based on the acquired frames using Fourier decomposition to extract the signal from biological noise using an analysis software package for continuous recording of optical intrinsic signals (VK Imaging, USA) (16). The ODI was then calculated for every pixel within the binocularly responding region based on the formula (C-I)/(C+I), where “C” refers to the response magnitude of the contralateral eye and “I” to that of the ipsilateral eye. For each animal, several ODIs were collected and then averaged. Positive ODI values represent contralateral dominance, negative represent ipsilateral dominance, while ODI values of 0 correspond to equally strong contralateral and ipsilateral eyes.

## **In vivo electrophysiology**

Before electrophysiological recordings, PV-cre mice underwent transparent skull surgery and optoTrkB was injected as described above. Under 15% urethane anesthesia (in PBS), a hole for a 16 channel optrode (A1x16-10mm-100-177-OA16LP, NeuroNexus, Ann Arbor, MI) was drilled next to the injection site and a hole for a ground electrode next to lambda. The animals were head-fixed and the optrode was covered with dye and slowly inserted into the V1 to a depth of about 2200 µm. Local field potential data were recorded from the binocular region of V1 of the optoTrkB transfected area using a Smartbox (NeuroNexus, Ann Arbo0r, MI) at a sampling rate of 20 kHz during acquisition. After collecting a 20 minute baseline, optoTrkB was stimulated for 30 seconds with blue LED light and recorded for 2h. Finally, the animals were transcardially perfused and the brains fixed with 4% PFA. To confirm the position of the electrodes and co-localization with optoTrkB, 250 µm coronal slices were cut on a vibratome.

The digitized data were first offline notch filtered at 50 Hz by the Python RHD2000 interface provided by Intan. Afterwards, the data were band-pass filtered between 4 and 150 Hz, using a 2nd order Butterworth filter with zero-phase shift, and then downsampled to a sampling rate of 1 kHz. Data were then separated into 20-minute blocks including one baseline and five blocks after optotrack stimulation. The LFP for each electrode contact was normalized to zero and the underlying current source density (CSD) was calculated as the second spatial derivative, using previously published methods (18).

Time-frequency analysis of the CSD data was performed by wavelet filtering with 36 Morlet wavelets ranging from 4 to 112 Hz with log-constant spacing and with m = 6. Data from all electrodes that were identified to reside in areas with optoTrkB expression by the histological analysis was then averaged, and binned into non-overlapping 20 seconds bins. Morlet power spectra were estimated by computing the averaged magnitude over all bins of the 20-minute recording period per condition. For statistical analysis, broadband power in the 20-minute baseline period and the 20-minute period beginning 100 minutes after LED onset was compared. The effect size was measured using Cohen’s d. All data analyses and statistics were performed in Python (ver. 2.7) using SciPy (ver. 0.19.0), the NeuroDSP toolbox (ver. 2.0.1-dev)(19), and custom written scripts.

## **Electrophysiology in acute slices**

The brains of optoTrkB-infected PV-cre mice were dissected in darkness using red light illumination, and kept in the dark throughout the whole experiment. The brains, including brains of fluoxetine-treated WT and hPV-TrkB cKO mice, were dissected and immersed in ice-cold dissection solution containing (in mM): 124 NaCl, 3 KCl, 1.25 NaH_2_PO4, 1 MgSO_4_, 26 NaHCO_3_, 15 D-glucose, 9 MgSO_4_ and 0.5 CaCl_2_. The cerebellum and anterior part of the brain were removed and coronal 350µm brain slices of the V1 were cut on a vibratome (Leica Biosystems, Wetzlar, Germany). Slices were divided into two groups and allowed to recover for 30 min at 31-32°C in artificial cerebrospinal fluid (ACSF) containing (in mM): 124 NaCl, 3 KCl, 1.25 NaH_2_PO_4_, 1 MgSO_4_, 26 NaHCO_3_, 15 D-glucose, and 2 CaCl_2_ and bubbled with 5% CO_2_/95% O_2_.

OptoTrkB-transfected slices in one of the groups were acutely stimulated for 30 seconds with blue light after transferring them to the recording chamber, whereas the transfected slices in the other group were kept in darkness.

Field excitatory postsynaptic currents (fEPSPs) were recorded in an interface chamber (32°C) with ACSF-filled glass microelectrodes (2-4 MΩ) positioned within layer II/III of the V1 using an Axopatch 200B amplifier (Molecular devices, San Jose, CA). Electric stimulation (100 µsec duration) was delivered with a bipolar stimulation electrode placed at the border of the white matter (WM) and layer VI. Baseline synaptic responses were evoked every 20 seconds with a stimulation intensity that yielded a half-maximum response. After obtaining a 15 minute stable baseline, θ burst stimulation (TBS) (4 sweeps at 0.1 Hz, each sweep with 10 trains of 4 pulses at 100 Hz at 200 ms intervals) was delivered and field potentials in response to 0.05 Hz stimulation were recorded for additional 45 minute. WinLTP (0.95b or 0.96, [www.winltp](http://www.winltp).com) was used for data acquisition and analysis.

To measure intrinsic excitability, the brains of PV-cre mice transfected with the DIO-optoTrkB-IRES-TdT lentivirus or hPV-TrkB cKO and WT mice expressing TdTomato in PV cells were dissected as described above but cut in a protective NMDG ACSF (20) containing (in mM): 92 NMDG, 2.5 KCl, 1.25 NaH_2_PO_4_, 30 NaHCO_3_, 20 HEPES, 25 glucose, 2 thiourea, 5 Na-ascorbate, 3 Na-pyruvate, 0.5 CaCl_2_·4H_2_O and 10 MgSO_4_·7H_2_O, pH 7.3–7.4 The slices were allowed to recover at 32°C for 10 min, after which they were transferred to modified ACSF containing (mM): 92 NaCl, 2.5 KCl, 1.25 NaH_2_PO_4_, 30 NaHCO_3_, 20 HEPES, 25 glucose, 2 thiourea, 5 Na-ascorbate, 3 Na-pyruvate, 2 CaCl_2_·4H_2_O and 2 MgSO_4_·7H_2_O , pH 7.3-7.4 for storage. Recordings were done in submerged chamber perfused with normal ACSF (32°C).

Whole cell patch clamp recordings from the TdT expressing PV cells were obtained under visual guidance under ambient light with glass microelectrodes (3-5 MΩ) filled with a low Cl^-^-filling solution containing (in mM): 135 K-gluconate, 10 HEPES, 2 KCl, 2, Ca(OH)_2_, 5 EGTA, 4 Mg-ATP, 0.5 Na-GTP using a Multiclamp 700A amplifier (Axon Instruments, USA). Uncompensated series resistance (Rs) was monitored by measuring the peak amplitude of the current response to a 5 mV step. Only experiments where Rs < 30 MΩ, and with < 20 % change in Rs during the experiment, were included in analysis.

Intrinsic excitability was measured in current clamp mode by injecting currents ranging from -50 to 600 pA for 600 ms in 50 pA steps from the resting membrane potential of -60/-70 mV. The recordings were analyzed in Clampfit (Molecular Devices, San Jose, CA) programs. A minimum of three action potentials (AP) were averaged for analysis of AP half-width (10^th^ AP at 200pA injected from rheobase). sEPSCs were recorded under voltage clamp at -70 mV and analyzed in Clampfit (Molecular Devices, San Jose, CA, USA). The threshold for detection of inward sEPSC events was three times the baseline noise level, and all detected events were verified visually.

Whole cell patch clamp recordings from layer II/III pyramidal neurons were obtained under visual guidance under ambient light with glass microelectrodes (4-5 MΩ) filled with a low Cl-filling solution containing (in mM): 136 Cs-MeSO_3_, 10 HEPES, 2 NaCl, 0.5 EGTA, 4 Mg-ATP, 0.3 Na-GTP, 5 QX314. Electric stimulation (100 μsec duration) was delivered with a bipolar stimulation electrode placed at the border of the white matter (WM) and layer VI. Synaptic responses were evoked every 30 seconds with a stimulation intensity that yielded a half-maximum response. Excitatory synaptic responses were recorded at -70 mV, and inhibitory synaptic responses were recorded at 0 mV holding potential. The disynaptic nature of the IPSC was confirmed by a 10.3 ms delay in average of onset compared to the monosynaptic EPSC. WinLTP (0.95b or 0.96, www.winltp.com) was used for data acquisition and analysis.

# **Sample collection**

For collecting tissue samples for qPCR and Western Blot experiments, the brains of lentiviral optoTrkB infected PV-cre mice were dissected and immersed in ice-cold dissection solution containing (in mM): 124 NaCl, 3 KCl, 1.25 NaH_2_PO_4_, 1 MgSO_4_, 26 NaHCO_3_, 15 D-glucose, 9 MgSO_4_ and 0.5 CaCl_2_. The V1 was dissected and incubated at 31-32°C in artificial cerebrospinal fluid (ACSF) containing (in mM): 124 NaCl, 3 KCl, 1.25 NaH_2_PO_4_, 1 MgSO_4_, 26 NaHCO_3_, 15 D-glucose, and 2 CaCl_2_ and bubbled with 5% CO_2_/95% O_2_. The tissue samples were either immediately collected in NP lysis buffer and homogenized (control), or stimulated with blue LED light for 30 seconds and collected and homogenized after 15 minutes, 30 minutes or 60 minutes. All of the procedures were done in dark conditions. The samples were further used for qPCR and Western Blot analysis. In order to obtain slices for immnohistochemistry, PV-cre mice infected with lentiviral DIO-optoTrkB in the VC were stimulated with blue LED light (470nm) through the transparent skull for 30 seconds, and the mice were perfused one hour after the stimulation (acute samples). For chronic samples the infected mice were stimulated in accordance with the OD experiment (twice daily for 30 seconds at 8-10 am and 4-6 pm during 7 days). Then the mice were perfused 3hr after the last stimulation.

# **Western Blot**

The samples were centrifuged (16000 g, 15 min at +4°C) and the supernatant was used to measure the protein concentrations using the Lowry method (Biorad DC protein assay, BioRad, Richmond, CA) (Lowry et al, 1951). The samples were separated in a SDS-PAGE (2-4% gradient gel, NuPage™; ThermoFisher Scientific, Hvidovre, Denmark) and blotted to a PVDF membrane (300 mA, 1 h, + 41°C). The membranes were washed in Tris Buffer Solution with 0,001% Tween ®20 (TBST), blocked in TBST with 3% BSA for 1 hour at room temperature and incubated in primary antibody solutions (in TBST with 3% BSA) directed against: phosphorylated and non-phosphorylated forms of TrkB (Y816, Y705/6, Y515) and CREB at +4°C for overnight. After washing in TBST, the membranes were further incubated in secondary antibody solutions (TBST with 5% Non-Fat Dry Skinned Milk and Horseradish Peroxidase conjugated secondary antibodies Goat Anti-Rabbit/Mouse, 1:10000) for 2 hours at room temperature. After washing with TBST and rinsing with PBS, secondary antibodies were visualized by an electrochemiluminescence kit (ECL plus, ThermoFisher Scientific, Hvidovre, Denmark) according to the manufactures instruction, and detected using a FUJIFILM LAS-3000 dark box.

# **qPCR**

RNA was purified from the lysate following the manufacturer’s protocol using a combined protocol of QIAzol® (Qiagen , Hilden, Germany) and NucleoSpin® (Macherey-Nagel , Düren, Germany). Briefly, the aqua layer was isolated after Qiazol and chloroform extraction. The RNA was washed in 100% ethanol and the DNA was digested in the spin columns. The purified RNA was then reverse transcribed to cDNA using Maxima First Strand cDNA Synthesis Kit (ThermoFisher Scientific, Hvidovre, Denmark). The amount of cDNA synthesized from the target mRNA was quantified by real-time PCR (qPCR) in triplicate and the values were averaged for each sample. The following primers were used to amplify specific cDNA regions of the transcripts of interest:

Kv3.1 (5’-AGAGATTGGCACTCAGTGACT-3’ and 5’-TTGTTCACGATGGGGTTGAAG-3’), Kv3.2 (5’-AGGCTATGGGGATATGTACCC-3’ and 5’-TGCAAAATGTAGGCGAGCTTG-3’), PV (5’-TGTCGATGACAGACGTGCTC-3’ and 5’-TTCTTCAACCCCAATCTTGC-3’), FosB_(5’-AGTTTGTCCTGGTGGCCC-3’ and 5’-GGATGTTGACCCTGGCAAAT-3’), and Syt2 (5’-AGAACCTGGGCAAATTGCAGT-3’ and 5’-CCTAACTCCTGGTATGGCACC-3’).

## **Immunohistochemistry**

Animals were transcardially perfused with PBS followed by chilled 4% paraformaldehyde (PFA) in PBS. Brains were removed under ambient light and left for fixation in 4% PFA overnight at +4 °C. For cutting, the brains were embedded in 3% agar and cut into 40 μm coronal visual cortical sections using a vibratome (Leica Biosystems, Wetzlar, Germany). After washing with PBST (1x PBS and 0.2% TritonX100), the sections were incubated in 10% donkey serum (Vector Laboratories, UK) and/or 10% goat serum and 3% Bovine Serum Albumin (BSA) (Sigma-Aldrich, Steinheim, Germany) in PBST for 30 minutes at room temperature. Next, the sections were incubated with the following antibodies: 1) guinea pig anti-parvalbumin (1:1000) (Synaptic Systems, Göttingen, Germany), 2) biotinylated lectin from Wisteria floribunda (WFA; 1:200) (Sigma-Aldrich, Steinheim, Germany), and 3) rabbit anti-phospho Kv3.1 (1:100) (Phosphosolutions, Aurora, CO), (4) rabbit anti-Glutamate Receptor 2 and 3 (GluR2/3) (1:100) (AB1506, Chemicon), and (5) mouse anti-Syt2 (1:125) (DSHB, Iowa city, IA) overnight at +4° C. After washing in PBST, the samples were further incubated in secondary antibody dilutions, including 1) goat anti-guinea pig conjugated with Alexa Fluor647/546 (1:1000) (Thermofisher Science, Waltham, MA), 2) streptavidin conjugated with Alexa Fluor488 (1:1000) (Thermofisher Science, Waltham, MA), 3) Goat anti-rabbit conjugated with Alexa Fluor 647 (1:1000) (Life technologies, Carlsbad, CA), 4) Donkey anti-rabbit conjugated with Alexa 488 (1:400) (Thermofisher Science, Waltham, MA), or 5) Donkey anti-Mouse IgG conjugated with Alexa 647 (1:400) (Thermofisher Science, Waltham, MA) for 1-2 hours at room temperature protected from light. After final washing in PBS, the sections were transferred to 0.1M PB with gelatin, mounted on glass slides and covered with DAKO mounting medium (Sigma Aldrich, Steinheim, Germany).

## **Image acquisition and analysis**

Quantitative analysis of immunostainings was performed blind. Images were taken from the V1 according to the mouse brain atlas.

Laser scanning confocal microscopy was used to detect PV positive (PV^+^), PNN positive (PNN^+^), double positive (PV^+^PNN^+^) and pKv3.1-positive cells. Images were obtained using a confocal microscope LSM 700 (Carl Zeiss) equipped with a 10× objective lens (10x Plan-Apochromat 10x/0.45, Carl Zeiss) and imaging Software ZEN 2012 lite (Zeiss, Vantaa, Finland). From each section, a z-stack containing at least 10 consecutive images was obtained. A minimum number of 3 sections per animal were imaged using the same microscope and the same camera settings for all samples. Image processing was done using Fiji software (https://fiji.sc/) (21). All images in each z-stack were analyzed and the number of cells was averaged per z-stack.

To determine the PV cell populations, frequency distribution analyses were performed on PV intensities taken from non-stimulated optoTrkB samples or control WT samples to serve as reference group. The PV cell populations were defined as low PV (0-8000 a.u.), intermediate-low PV (int-low PV, 8000-16000 a.u.), intermediate-high PV (int-high, 16000-24000 a.u.) and high PV (24000-36000 a.u.) expressing cells and these criteria were applied to the light stimulated samples.

For imaging analysis on Syt2, images were obtained using a confocal microscope LSM 700 with a 63× objective lens (Plan-Apochromat 63x/1.0, Carl Zeiss) and 4x digital zoom. Images of GluR2/3 and Syt2 positive cells were obtained with z-stack with a 0.448 µm interval and taken at least 10 images in one group. Syt2 puncta were analyzed throughout the z-stack using a fire scale and only white puncta were counted. Intensity of Syt2 was analyzed by Fiji (21) on one representative layer of each cell, which has the largest area of Syt2 expression. All analyses were performed double-blinded.

## **Statistical analysis**

All statistical graphs were generated using Graphpad Prism v.6.07 (GraphPad Software, San Diego, CA). Unpaired t-Test, Two-way and one-way ANOVA followed by Tukey’s or or Holm-Sidak’s post hoc tests were also performed using Graphpad Prism v.6.07. Distributions of all data were checked, and normally distributed data was analyzed parametrically. Otherwise data was analyzed with non-parametric ways. The sample size was determined based on our previous experience. The significant level was set to 0.05 (P value) and all bar-graph were presented as means ± s.e.m.

**Reference**

1. Hippenmeyer S, Vrieseling E, Sigrist M, Portmann T, Laengle C, Ladle DR, et al. A Developmental Switch in the Response of DRG Neurons to ETS Transcription Factor Signaling. PLoS Biol. 2005;3(5):e159.
2. Minichiello L, Korte M, Wolfer D, Ku R, Unsicker K, Cestari V, et al. Essential Role for TrkB Receptors in Hippocampus-Mediated Learning. Neuron. 1999;24(2):401-14.
3. Madisen L, Zwingman TA, Sunkin SM, Oh SW, Zariwala HA, Gu H, et al. A robust and high-throughput Cre reporting and characterization system for the whole mouse brain. Nat Neurosci. 2010;13(1):133-40.
4. Lehmann K, Löwel S. Age-Dependent Ocular Dominance Plasticity in Adult Mice. PLoS One. 2008;3:e3120
5. Chang KY, Woo D, Jung H, Lee S, Kim S, Won J, et al. Light-inducible receptor tyrosine kinases that regulate neurotrophin signalling. Nat Commun. 2014;5(1):1–10.
6. Kennedy MJ, Hughes RM, Peteya LA, Schwartz JW, Ehlers MD, Tucker CL. Rapid blue-light–mediated induction of protein interactions in living cells. Nat Methods. 2010;7(12):973–5.
7. Fenno LE, Mattis J, Ramakrishnan C, Hyun M, Lee SY, He M, et al. Targeting cells with single vectors using multiple-feature Boolean logic. Nat Methods. 2014;11(7):763–72.
8. Kügler S, Meyn L, Holzmüller H, Gerhardt E, Isenmann S, Schulz JB, et al. Neuron-Specific Expression of Therapeutic Proteins: Evaluation of Different Cellular Promoters in Recombinant Adenoviral Vectors. Mol Cell Neurosci. 2001;17(1):78–96.
9. Lee G, Saito I. Role of nucleotide sequences of loxP spacer region in Cre-mediated recombination. Gene. 1998;216(1):55–65.
10. Dittgen T, Nimmerjahn A, Komai S, Licznerski P, Waters J, Margrie TW, et al. Lentivirus-based genetic manipulations of cortical neurons and their optical and electrophysiological monitoring in vivo. Proc Natl Acad Sci. 2004;101(52):18206–11.
11. Shaner NC, Campbell RE, Steinbach PA, Giepmans BNG, Palmer AE, Tsien RY. Improved monomeric red, orange and yellow fluorescent proteins derived from Discosoma sp. red fluorescent protein. Nat Biotechnol. 2004;22(12):1567–72.
12. Weber K, Mock U, Petrowitz B, Bartsch U, Fehse B. Lentiviral gene ontology (LeGO) vectors equipped with novel drug-selectable fluorescent proteins: new building blocks for cell marking and multi-gene analysis. Gene Ther. 2010;17(4):511–20.
13. Hioki H, Kameda H, Nakamura H, Okunomiya T, Ohira K, Nakamura K, et al. Efficient gene transduction of neurons by lentivirus with enhanced neuron-specific promoters. Gene Ther. 2007;14(11):872–82.
14. Tiscornia G, Singer O, Verma IM. Production and purification of lentiviral vectors. Nat Protoc. 2006;1(1):241–5.
15. Steinzeig A, Molotkov D, Castrén E. Chronic imaging through “transparent skull” in mice. Homberg J, editor. PLoS One. 2017;12(8):e0181788.
16. Kalatsky VA, Stryker MP. New paradigm for optical imaging: temporally encoded maps of intrinsic signal. Neuron. 2003;38(4):529–45.
17. Cang J, Kalatsky V a, Löwel S, Stryker MP. Optical imaging of the intrinsic signal as a measure of cortical plasticity in the mouse. Vis Neurosci. 2005;22(5):685–91.
18. Voigt MB, Kral A. Cathodic-leading pulses are more effective than anodic-leading pulses in intracortical microstimulation of the auditory cortex. J Neural Eng. 2019;16(3).
19. Cole S, Donoghue T, Gao R, Voytek B. NeuroDSP: A package for neural digital signal processing. J Open Source Softw. 2019;4(36):1272.
20. Ting JT, Daigle TL, Chen Q, Feng G. Acute Brain Slice Methods for Adult and Aging Animals: Application of Targeted Patch Clamp Analysis and Optogenetics. In Humana Press, New York, NY; 2014. p. 221–42.
21. Schindelin J, Arganda-Carreras I, Frise E, Kaynig V, Longair M, Pietzsch T, et al. Fiji: An open-source platform for biological-image analysis. Nat Methods. 2012;9(7): 676-82.

**Supplemental Figure Legends**

**Supplementary Figure 1. Confirmation of inversion of DIO-optoTrkB by cre-recombinase**

(a) The plasmid to produce lentivirus DIO-optoTrkB. (b) Confirmation of inversion of DIO-optoTrkB by cre-recombinase. HEK293 cells were co-transfected with pLenti-DIO-optoTrkB and pLenti-CAG-GFP-IRES-CRE or pAAV-EF1a-Cre, which express cre-recombinase. PCR analysis revealed that DIO-optoTrkB was inverted after co-transfection with cre but not when expressed alone. (c) The plasmid to produce DIO-optoTrkB-IRES-TdTomato. (d) Confirmation of inversion of DIO-optoTrkB-IRES-TdTomato by cre-recombinase. HEK293 cells were transfected with either or both pLenti-CAG-GFP-IRES-CRE and pLenti-optoTrkB-IRES-TdTomato. Only co-transfected cells expressed TdTomato demonstrating that the expression is cre-dependent. hSyn, human Synapsin promoter; oPHR, optimised Photolyase homology region; fTrkB, full-length TrkB.

**Supplementary Figure 2. Western Blot analysis of optoTrkB expressed in PV interneurons.**

Western Blot analysis of V1 of PV-cre mice infected with DIO-optoTrkB without light stimulation (Control) and 15 minutes, 30 minutes and 60 minutes after light stimulation. (a) Representative Western Blots of Y816, Y515, Y706 and CREB. Acute stimulation of optoTrkB with light results in increased phosphorylation of tyrosine sites: (b) Y816, (c) Y515, (d) Y706 and (e) phosphorylation of CREB.

**Supplementary Figure 3.**

(a) Shift of ocular dominance experiments without infections of optoTrkB lentivirus

PV-cre mice without optoTrkB expression stimulated with blue light twice daily during 7 days of MD show no shift in ocular dominance (p = 0.2852, unpaired t-test) (N=6). (b) qPCR of Kv3.1 mRNA with V1 samples after optoTrkB activation in PV interneurons. OptoTrkB reduces the expression of Kv3.1 60 minutes after stimulation (one-way ANOVA followed by Holm-Sidak post-hoc test: control vs. 60 min, p = 0.0895, Control (non-stimulated, n = 4), 15 minutes (n = 4), 30 minutes (n = 4) and 60 minutes (n = 3) after light stimulation).

**Supplementary Figure 4. Patch clamp recordings after fluoxetine treatment and optoTrkB activation**

(a) Representative traces of electrophysiological recordings. Representative traces for recording of intrinsic excitability (150 pA step, top row), AP (2nd row) and spontaneous synaptic currents (sEPSCs) (bottom row) in WT mice treated with water (first column), WT mice treated with Flx (2nd column), CKO mice treated with water (3rd column) and CKO mice treated with Flx (last column). (b) frequency and amplitude (c) of sEPSC and (d) frequency and (e) amplitude of sIPSC amplitudes are unchanged after chronic fluoxetine treatment in both genotypes. (f) Representative traces of electrophysiological recordings of intrinsic excitability (top row, 150 pA), AP (middle row) and sEPSC (lower row) in optoTrkB infected slices. (g) AP half-width, (h) frequency of sEPSC, (i) frequency and (j) amplitude of sIPSC are unchanged after optoTrkB activation.

**Supplementary Figure 5. Time course of LFP power difference between control and light**

Box plots for θ, α, β, and γ frequency bands show the difference between red- and blue-light-activated animals (Control, N = 4; Activated, N = 5) for each 20 minute recording session as a function of time after light stimulation. LFP power of each band gradually increased stronger during the 100 minutes after optoTrkB activation than after the red-light control.

**Supplementary Table 1.** **Measurement of light intensity of blue LED light and room light through a transparent skull with or without black painting**

**Supplementary Table 2.** **Input resistance is after optoTrkB activation and fluoxetine treatment in WT and hPV-TrkB CKO mice.**

(a) Input resistance is unchanged after optoTrkB activation. (b) No effect of fluoxetine treatment on input resistance in WT and hPV-TrkB CKO mice.
