# Supplementary figures and images for: Pharmacological and optical activation of TrkB in Parvalbumin interneurons regulate intrinsic states to orchestrate cortical plasticity
Source: Mol Psychiatry. 2021 Jul 28;26(12):7247–56. doi: 10.1038/s41380-021-01211-0 (PMC8872988; doi:10.1038/s41380-021-01211-0)

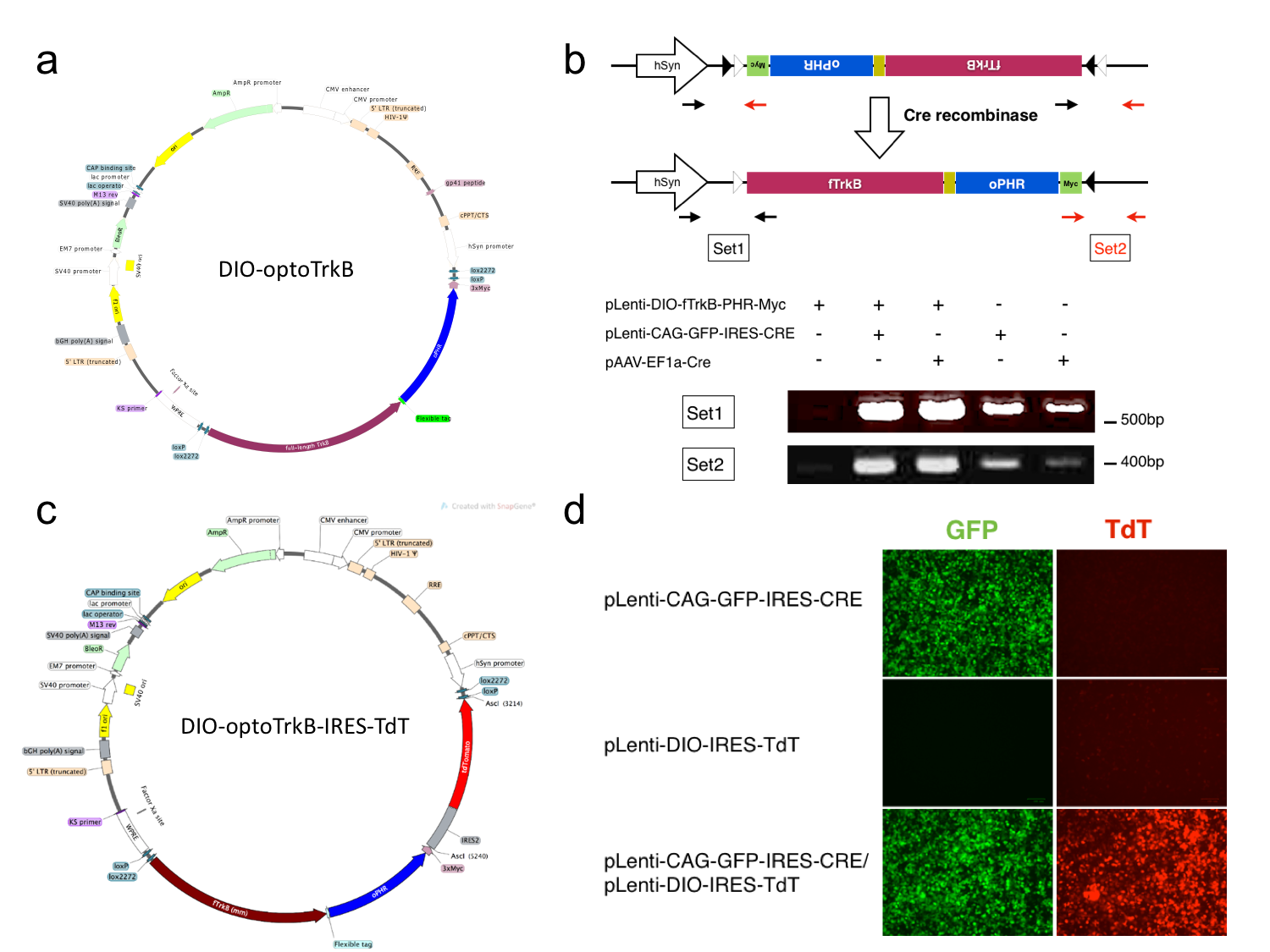

Supplement: Supplementary file 2 — Supplemental Figure 1 [file 41380_2021_1211_MOESM2_ESM.tif]

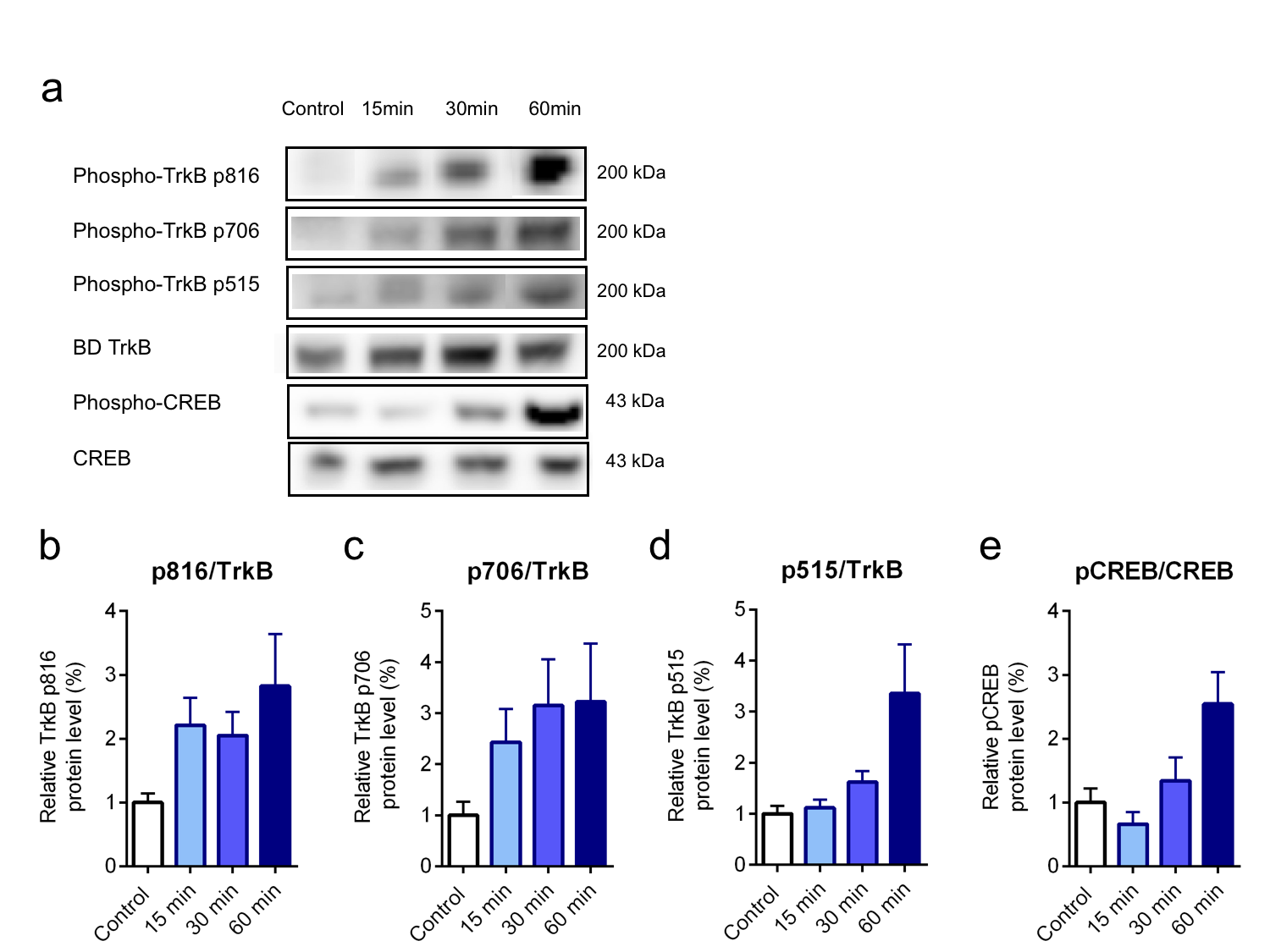

Supplement: Supplementary file 3 — Supplemental Figure 2 [file 41380_2021_1211_MOESM3_ESM.tif]

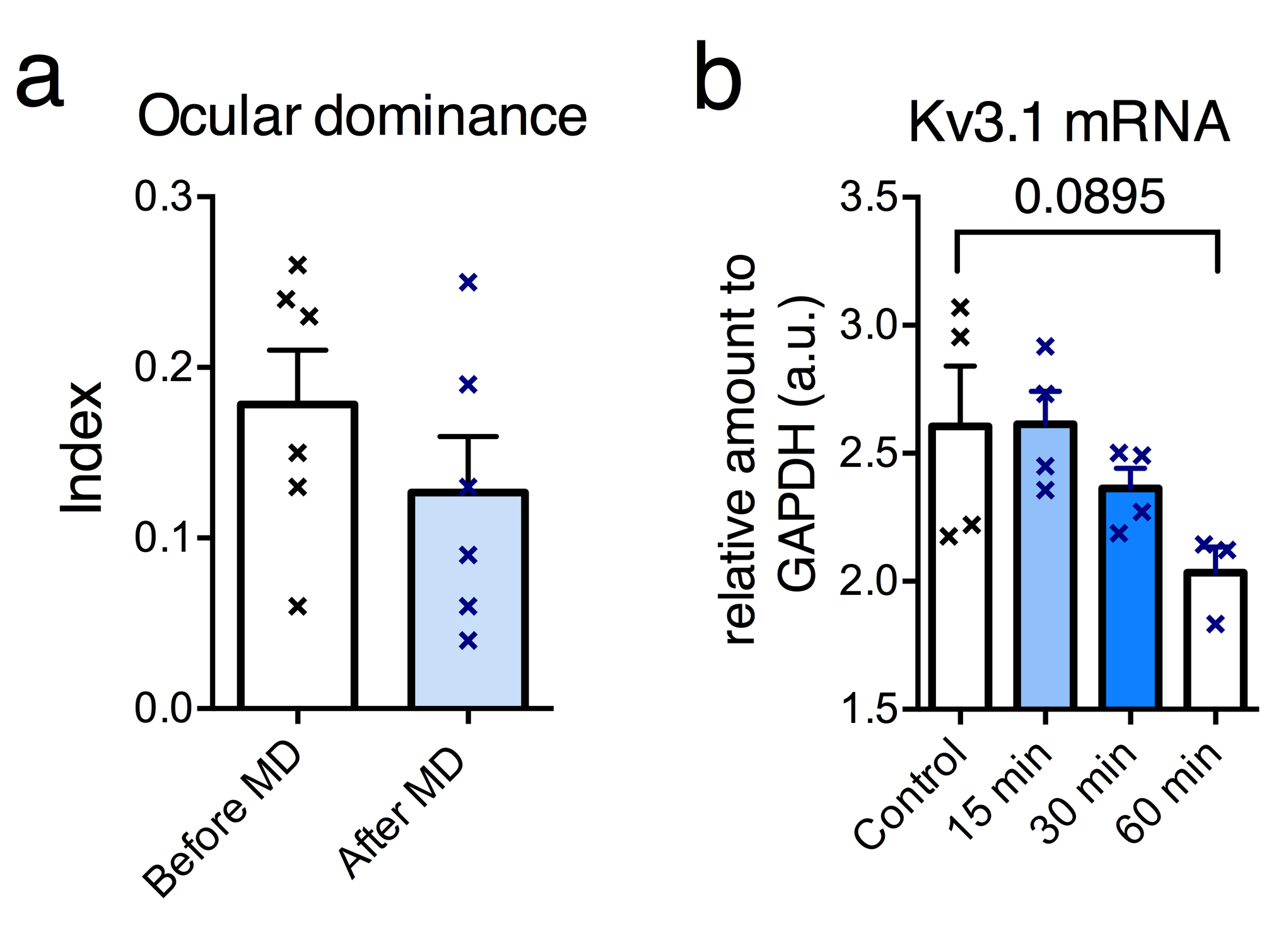

Supplement: Supplementary file 4 — Supplemental Figure 3 [file 41380_2021_1211_MOESM4_ESM.tif]

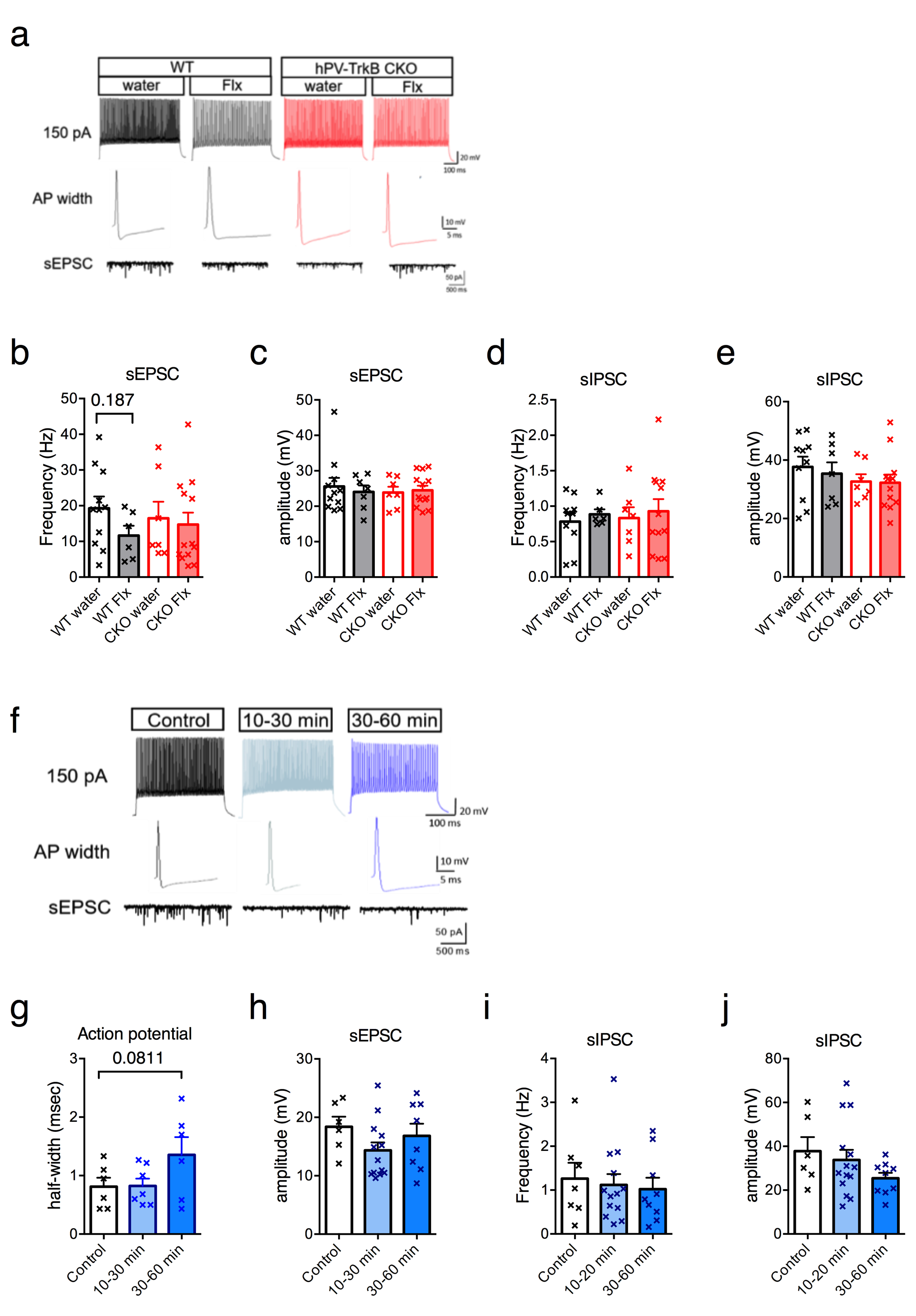

Supplement: Supplementary file 5 — Supplemental Figure 4 [file 41380_2021_1211_MOESM5_ESM.tif]

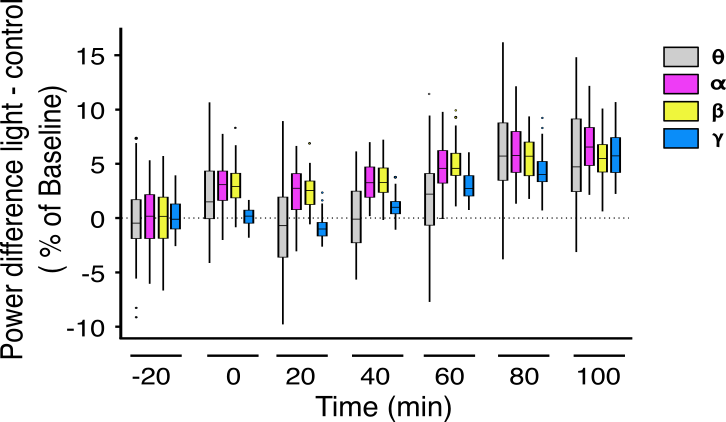

Supplement: Supplementary file 6 — Supplemental Figure 5 [file 41380_2021_1211_MOESM6_ESM.png]

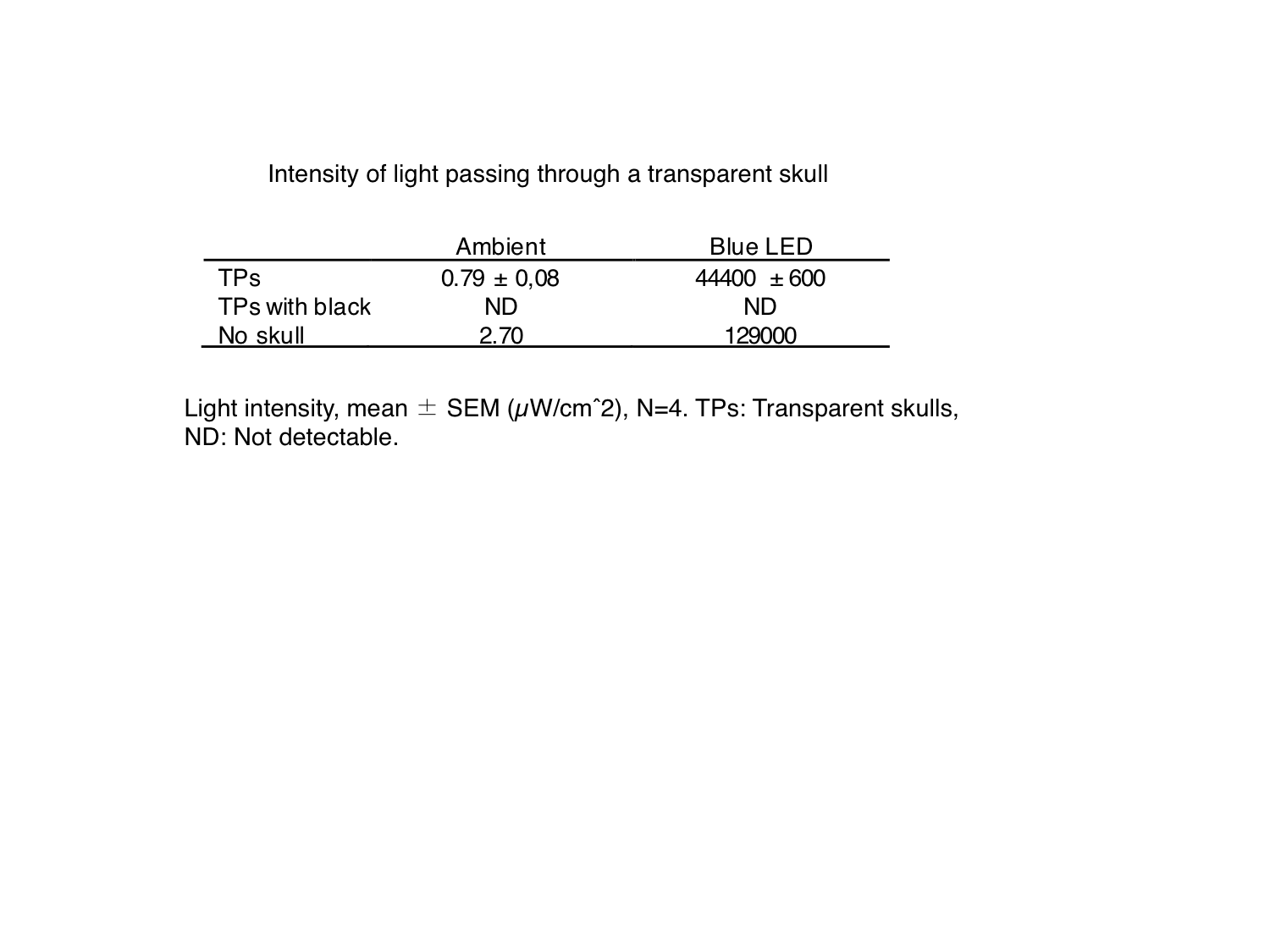

Supplement: Supplementary file 7 — Supplemental Table 1 [file 41380_2021_1211_MOESM7_ESM.tif]

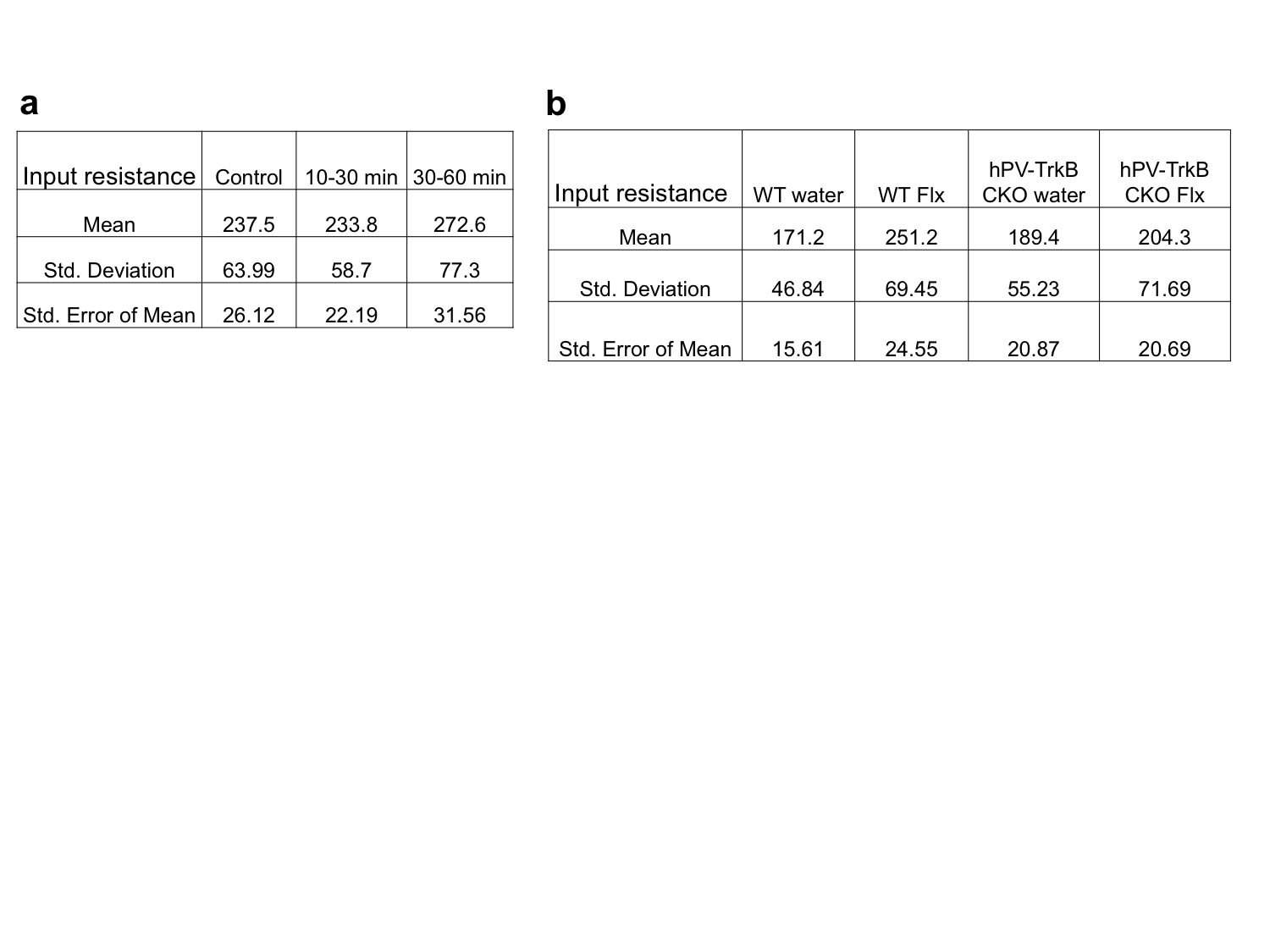

Supplement: Supplementary file 8 — Supplemental Table 2 [file 41380_2021_1211_MOESM8_ESM.tif]
